# Tuning Net Charge in Aliphatic Polycarbonates Alters Solubility and Protein Complexation Behavior

**DOI:** 10.1021/acsomega.1c02523

**Published:** 2021-08-26

**Authors:** Nicholas D. Posey, Yuanchi Ma, Michael Lueckheide, Julia Danischewski, Jeffrey A. Fagan, Vivek M. Prabhu

**Affiliations:** Materials Science and Engineering Division, Material Measurement Laboratory, National Institute of Standards and Technology, 100 Bureau Drive, Gaithersburg, Maryland 20899, United States

## Abstract

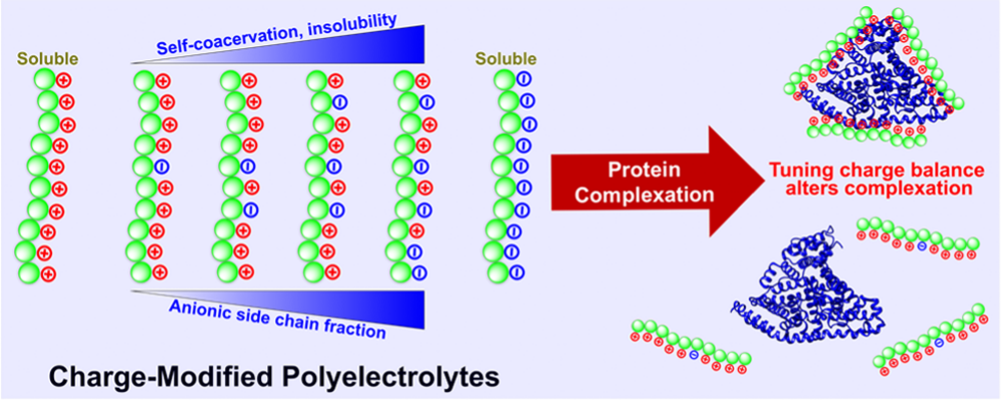

A synthetic strategy yielded polyelectrolytes and polyampholytes with tunable net charge for complexation and protein binding. Organocatalytic ring-opening polymerizations yielded aliphatic polycarbonates that were functionalized with both carboxylate and ammonium side chains in a post-polymerization, radical-mediated thiol–ene reaction. Incorporating net charge into the polymer architecture altered the chain dimensions in phosphate buffered solution in a manner consistent with self-complexation and complexation behavior with model proteins. A net cationic polyampholyte with 5% of carboxylate side chains formed large clusters rather than small complexes with bovine serum albumin, while 50% carboxylate polyampholyte was insoluble. Overall, the aliphatic polycarbonates with varying net charge exhibited different macrophase solution behaviors when mixed with protein, where self-complexation appears to compete with protein binding and larger-scale complexation.

## Introduction

Biodegradable polymers and aliphatic polycarbonates (APCs) are used in the development of drug delivery strategies for many types of therapeutics and biopharmaceuticals.^1,2^ Biopharmaceutical drugs include protein-based therapies that are an important segment of the pharmaceutical industry.^2−4^ Biomolecular protein drugs are complex cargo in delivery applications.^2,4−7^ Key contributors to this complexity are the anisotropic and amphoteric characters of protein cargo due to heterogeneous, charged surface patches.^6,8−10^ Although proteins exhibit an overall net charge at a given pH, they may display oppositely charged patches on the surface that interact with other charged macromolecules.^8,11,12^ Thus, charge is a critical physicochemical parameter when using proteins for therapeutics. Accordingly, charged polymers (polyelectrolytes) are often used for delivery applications to facilitate protein interactions.^13−18^ Charge complementarity or matching positive polyelectrolytes with negative peptides/proteins and vice versa is a typical approach for promoting desirable interactions or encapsulation.^19−22^

Hedrick, Yang, and co-workers have developed biodegradable APCs and delivered protein therapeutics among other types of cargo.^23−26^ APC backbones are advantageous for biomaterials because of their biodegradability^27^ and ease of post-polymerization modification via “click” reactions, such as thiol–ene.^28−30^ APCs are seldom synthesized with ampholytic character, with only two syntheses reported.^31,32^ However, alternate synthetic schemes for amphoteric or ampholytic materials and their protein interactions have been explored.^1,33−36^

Charge optimization of biomaterials via APCs is attractive due to an available variety of post-polymerization modification strategies amenable for novel biomedical applications. Thus, in this work, we detail the synthesis of charged APCs with statistical mixtures of positively and negatively charged side chains based on ammonium and carboxylate moieties, respectively. These polyampholytes with net charge have aliphatic polycarbonate backbones, relevant to biomedical applications. Polyampholyte self-coacervation was observed and will be described. Dynamic light scattering (DLS) and small-angle neutron scattering (SANS) techniques provide a non-invasive approach to assess the ability of the charged polymers to bind to proteins and identify regimes of large-scale aggregation from the nanometer to micrometer length scales. Other techniques also showed that introducing net charge altered solution behaviors. Our results will be described within the broader context of studies of polymer/protein interactions.^37,38^

## Experimental Section

### Materials

Methanol, dichloromethane (CH_2_Cl_2_), chloroform (CHCl_3_), butylated hydroxytoluene (BHT) stabilized, and inhibitor-free tetrahydrofuran (THF) were obtained from Fisher Scientific. Diethyl ether was purchased from Acros Organics (purity >99%, stabilized with BHT). Ethanol (anhydrous) was purchased from The Warner Graham Company. Isopropanol (anhydrous) was purchased from J.T. Baker. Deuterium oxide (D_2_O, 99.9 D atom %), deuterated methanol (MeOD-*d*_4_), deuterated dimethyl sulfoxide [DMSO-*d*_6_, 99.9 D atom % with and without tetramethylsilane (TMS)], and deuterated chloroform (CDCl_3_ 99.8 D atom % with TMS) were obtained from Cambridge Isotope Laboratories. All were used as received without further purification or alteration unless stated otherwise. Ultrapure water with a resistivity of 18.2 MΩ cm^–1^ from a Milli-Q apparatus was used throughout this study.

The following reagents at the specified concentrations were used to make 1× phosphate buffer solution (PBS): 137 mmol/L sodium chloride (NaCl, Sigma-Aldrich), 2.7 mmol/L potassium chloride (KCl, Sigma-Aldrich), 8 mmol/L dibasic disodium monohydrogen phosphate (Na_2_HSO_4_, Amresco), and 2 mmol/L monobasic potassium dihydrogen phosphate (KH_2_PO_4_, Amresco). The pH of the buffer was adjusted to 7.4 using a non-standardized high-pH, basic aqueous solution of sodium hydroxide (NaOH, Merck). The pH of the buffer was monitored with a common digital pH meter calibrated on the same day with buffered solutions. Deuterated 1× PBS was made using the same salt concentrations but with D_2_O (pH 7.49).

The following reagents were used for synthesis: 3,5-bis(trifluoromethyl)phenyl isocyanate (98%), cyclohexylamine (>99.9%), 5-methyl-5-allyloxycarbonyl-1,3-dioxan-2-one (97%), potassium methoxide (95%), 2-hydroxy-4′-(2-hydroxyethoxy)-2-methylpropiophenone (98%), 3-mercaptopropionic acid (MPA) (>99%), cysteamine hydrochloride (98%), benzoic acid (>99.5%), and triethylamine (TEA) (>99.5%). All except TEA (Fluka) were purchased from Sigma-Aldrich and used without further purification.

#### Equipment

Polymerizations were conducted in an argon-filled glovebox from mBraun equipped with oxygen and water sensors, a recirculation and regeneration reactor to remove water and oxygen, and an internal freezer with temperature control containing a chilled aluminum block with vial-sized, bored holes. Centrifugation of polymer precipitates to recover purified aliphatic polycarbonate was performed with a Becton Dickinson, Dynac 420101, 4 hole—50 mL rotor, max acceleration ≈1500 g, using 50 mL polypropylene Falcon tubes. A twin bulb Blak-Ray ultraviolet (UV) benchtop lamp (115 V, 15 W, λ = 365 nm) was used for all photoinitiated reactions. Spectra/Por Biotech regenerated cellulose dialysis membranes (a molar mass cutoff of 3500 kDa) were obtained from Spectrum.

### Instrumentation and Conditions

^1^H NMR spectra were recorded at 600 MHz using an UltraShield AVANCE II 600 MHz Bruker spectrometer equipped with a broadband inverse (BBI) room-temperature probe and Sample Xpress automatic sampler at 25 °C. ^1^H NMR spectra were analyzed in TopSpin 3.6.1 from Bruker Biospin GmbH and/or MestReNova version 6.1.0-6224 by Mestrelab Research. Proton chemical shifts (δ) were reported in ppm and referenced to either TMS (0.0 ppm) or DMSO (2.5 ppm) for DMSO-*d*_6_ samples. *J*-coupling constants (*J*) were calculated in Hz and reported where applicable. Integrations are reported as the number of H’s rounded to the nearest whole number. Peaks and splitting patterns were identified visually or by MestReNova software using the following abbreviations: s (singlet), d (doublet), dd (doublet of doublets), ddd (doublet of doublet of doublets), t (triplet), tt (triplet of triplets), dt (doublet of triplets), ddt (doublet of doublet of triplets), q (quartet), p (pentet/quintet), m (multiplet), comp (short for complex, indicating overlapping multiplets of magnetically non-equivalent protons), and br (broad). A Magritek Spinsolve benchtop 80 MHz NMR (Carbon model) with Spinsolve Software version 1.15.1 was used to check reaction conversion.

Size exclusion chromatography (SEC) was performed with a Tosoh EcoSEC outfitted with a differential refractive index detector in series with a Wyatt Dawn Heleos II multiangle (18 angles) light scattering detector and a Wyatt Viscostar III differential viscometer. THF, inhibited with BHT, was used as the eluent flowing at 1 mL/min. Two mixed-pored TSK Gel GMH_HR_-H columns in series were used as the stationary phase. The SEC system was operated at 35 °C. UV–vis spectra were recorded in quartz cuvettes using a Cary 5000 UV–vis–NIR spectrophotometer.

### Synthesis

#### Organourea Catalyst Synthesis (**2**)

Previously published procedures were followed to synthesize the urea catalyst.^1^ In brief, the entirety of a vial of 3,5-bis(trifluoromethyl)phenyl isocyanate [1 g, 3.92 mmol, 1 equivalent (equiv)] was dissolved in 5 mL of anhydrous, uninhibited THF from a solvent purifier. The solution was transferred via a syringe through a septum into a flame-dried flask under argon with a Teflon stir bar. Cyclohexylamine (≈0.5 mL, ≈4.37 mmol, ≈1.12 equiv) was transferred dropwise through the septum into the THF solution while stirring. After 20 to 30 min of reaction time at room temperature under argon, the septum was removed, and THF was evaporated. The flask was placed in a 40 °C water bath to remove residual cyclohexylamine and residual THF in vacuo. After the mixture became solid in vacuo, it was ground into a powder and dried again in vacuo. After additional drying, the obtained power was ground further and rinsed multiple times with dichloromethane (CH_2_Cl_2_) in a glass funnel with a fine-grain, sintered glass disc. The mass yield was 57.8%, and ^1^H NMR (proton nuclear magnetic resonance) confirmed the correct structure in accordance with the literature (Figure S1).^39^

#### APC Precursor (**3**)

**Scheme 1:**
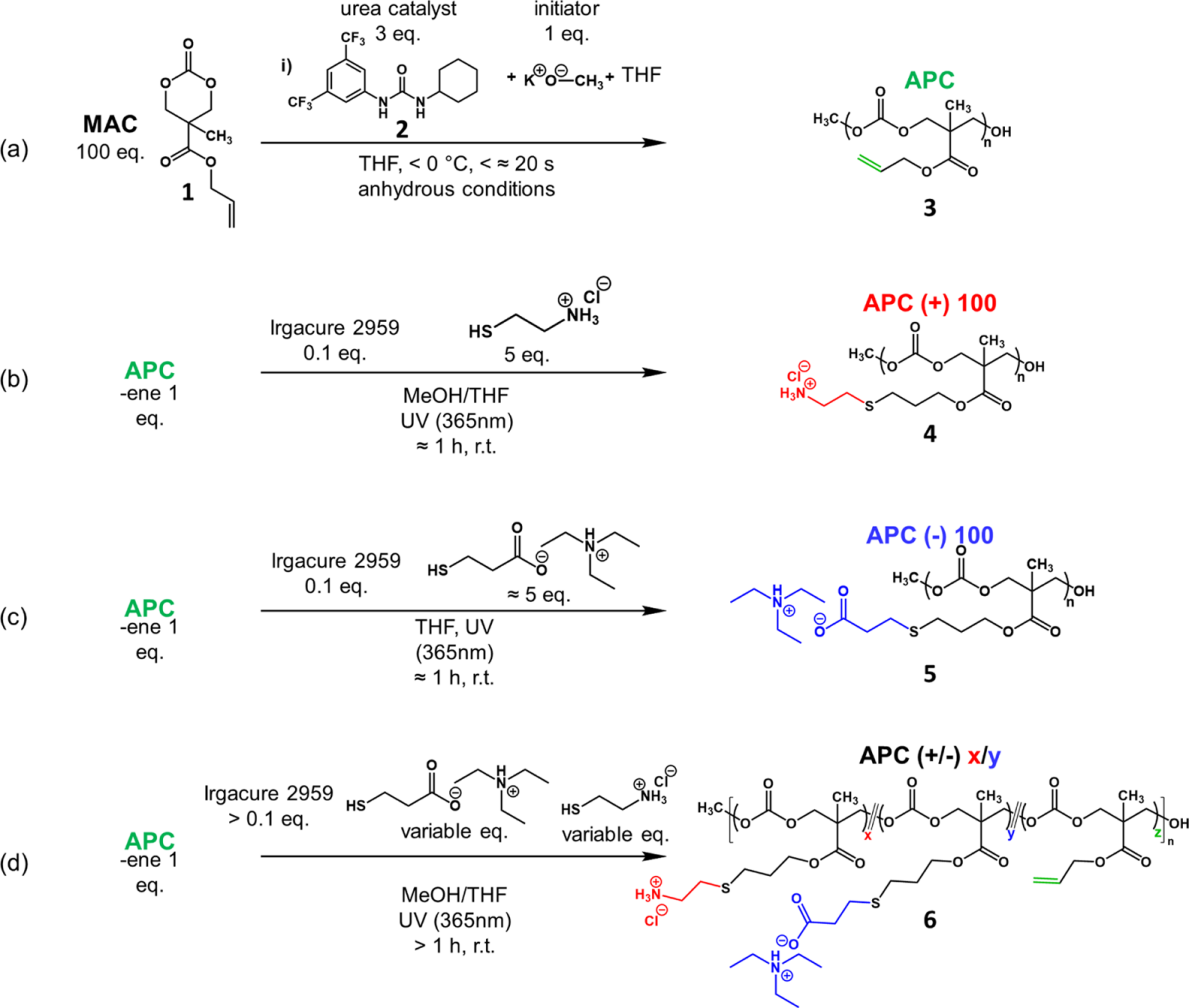
Synthesis of Charged APCs with Tunable Side-Chain Compositions

Scheme 1 presents the overall route of the synthesis, starting from a commercially available monomer, 5-methyl-5-allyloxycarbonyl-1,3-dioxan-2-one (MAC, **1**).^40^ Inside a glovebox (mBraun, UNILAB), **1** (2112.3 mg, 10.55 mmol, 100 equiv) was dissolved in 15 mL of anhydrous THF in a vial previously flame-dried and placed into a −10 °C freezer for 1 h. In a separate flame-dried vial, with the stir bar added later, the initiator potassium methoxide (KOMe, 7.4 mg, 0.1055 mmol, 1 equiv) and the organo-urea catalyst **2** (112.3 mg, 0.3168 mmol, 3 equiv) were combined, dissolved in 5 mL of anhydrous THF, and stored at −10 °C for 1 h inside the glovebox. Separately, the polymerization quencher, benzoic acid, was massed (154.3 mg, >10 equiv) and dissolved in 0.9 mL of THF and set aside. Polymerization was initiated by transferring the catalyst and initiator solution into the monomer solution (slightly warmed by hand) via a syringe. The reaction was conducted under stirring for 3 to 5 s. The benzoic acid solution was then added immediately to quench the reaction, and the solution became translucent. ^1^H NMR of the terminated reaction aliquot indicated 96.4% monomer conversion.

Following the polymerization, the solution was filtered through a polytetrafluoroethylene syringe filter (0.45 μm pore size, Millipore). The filtrate was concentrated by rotary evaporation, precipitated into 45 mL of chilled methanol, and then centrifuged for (10 to 15) min to recover a clear, viscous liquid after decantation. The crude product was then re-dissolved in a minimum amount of chloroform and again precipitated by methanol. Three successive precipitations were performed before the viscous liquid containing pure **3** was dried in vacuo to remove excess solvents. The identity of **3** was confirmed by ^1^H NMR (600 MHz, Bruker UltraShield), as can be found in Figure S1 of the Supporting Information.

^1^H NMR (600 MHz, CDCl_3_, with TMS at a volume fraction of 0.03%, spectra referenced to 0.0 ppm): δ 5.88 (ddd, *J* = 22.7, 10.8, 5.6 Hz, 1H), δ 5.31 (dd, *J* = 17.2, 1.3 Hz, 1H), δ 5.24 (dd, *J* = 10.5, 1.0 Hz, 1H), δ 4.63 (d, *J* = 5.6 Hz, 2H), δ 4.35–4.26 (m, 5H), δ 1.27 (s, 3H).

#### APC Polyelectrolytes (**4**, **5**, and **6**)

The syntheses of all APC polyelectrolytes followed the same general procedures exemplified by the following, whereas any modifications or deviations are noted in Table S1.

#### APC(+)100 (**4**)

**3**(497.8 mg, 2.48 mmol of ene groups, 1 equiv), cysteamine HCl (1462.3 mg, 12.87 mmol, 5.2 equiv), and the photoinitiator 2-hydroxy-4′-(2-hydroxyethoxy)-2-methylpropiophenone (57.5 mg, 0.256 mmol, 0.1 equiv) were dissolved in a mixture of 7 mL of THF (inhibitor free) and 7 mL of methanol in a glass vial that was sealed at the top with a rubber septum. The vial was sparged with argon for at least 1 min. After the sparging needle was removed, the vial was propped on one side against a cork ring, aligned parallel and in between the UV bulbs (365 nm wavelength, 15 W). After irradiation for 1 h, the vial was cooled, and the solution was precipitated into cold ethanol to remove excess cysteamine HCl, followed by centrifugation to recover the pure product, which was further concentrated by evaporation and then dried in vacuo. To aid in the process of removing the trapped residual solvent, the vial was frozen/thawed in liquid nitrogen in cycles under high vacuum. The resulting APC(+)100 **4** was confirmed by ^1^H NMR (Figure 2a).

^1^H NMR (600 MHz, DMSO-*d*_6_, spectra referenced to DMSO residual peak 2.5 ppm, ene peaks detected in the baseline but not listed): δ 8.22 (s, br, 3H), δ 4.34–4.18 (m, 4H), δ 4.15 (t, *J* = 6.0 Hz, 2H), δ 2.95 (t, *J* = 7.4 Hz, 2H), δ 2.77 (t, *J* = 7.3 Hz, 2H), δ 2.58 (t, *J* = 7.1 Hz, 2H), δ 1.89–1.78 (m calculated, p visual, 2H (set)), δ 1.19 (s, br, 3H).

#### APC(−)100 (**5**)

**3**(250.3 mg, 1.240 mmol of ene group, 1 equiv) and the photoinitiator (28.0 mg, 0.125 mmol, 0.1 equiv) were dissolved in 2 mL of THF (inhibitor-free). In a separate vial, TEA (0.85 mL, 6.13 mmol, 4.9 equiv) was mixed with MPA (0.55 mL, 6.3 mmol, 5 equiv). The slightly cloudy mixture was vortexed and diluted with 0.25 mL of fresh THF to homogenize the solution. The solutions in the two vials were mixed, and the reaction was UV-irradiated for 1 h. The crude reaction mixture was precipitated in a 50 mL Falcon tube filled with chilled diethyl ether and then decanted to remove the supernatant. The resulting polymer was purified further by dialysis against methanol and vac-dried to remove excess solvents. The final dialyzed **5** was characterized by ^1^H NMR (Figure 2b).

^1^H NMR (600 MHz, DMSO-*d*_6_, spectra referenced to DMSO residual peak 2.5 ppm): δ 4.31–4.16 (m, 4H), δ 4.13 (t, *J* = 5.9 Hz, 2H), δ 2.71–2.67 (m, br, 1H (6H expected), TEA), δ 2.65 (t, *J* = 7.2 Hz, 2H), δ 2.53 (t, *J* = 7.1 Hz, 2H), δ 2.46 (t, *J* = 7.1 Hz, 2H), δ 1.81 (p, *J* = 6.4 Hz, 2H (set)), δ 1.17 (s, br, 3H), δ 1.02 (t, *J* = 7.2 Hz, 1H (12H expected), TEA).

#### APC(+/−)*x*/*y* (**6**)

These ampholytic APC polyelectrolytes were synthesized by a one-pot reaction of **3**, cysteamine HCl, the pre-mixed TEA plus MPA solution, and the photoinitiator, with various feed ratios. The workup procedures were similar to those of **4** and **5**, albeit the solvents used for precipitation may differ, depending on the solubility of the specific polyampholyte (Table S1). ^1^H NMR can be seen in Figure S3.

#### APC(+/−)90/10 Synthesis

^1^H NMR (600 MHz, DMSO-*d*_6_ with TMS at a volume fraction of 0.03%, spectra referenced to 0.0 ppm): δ 7.71 (br, 24H), δ 5.87 (ddd, *J* = 22.0, 10.3, 5.0 Hz, 1H (set)), δ 5.28 (d, *J* = 17.3 Hz, 1H), δ 5.20 (d, *J* = 10.5 Hz, 1H), δ 4.60 (d, *J* = 3.9 Hz, 1H), δ 4.33–4.18 (m, 44H), δ 4.15 (t, *J* = 6.1 Hz, 19H), δ 2.94 (t, *J* = 7.3, 20H), δ 2.74 (d, br, *J* = 5.0 Hz, 20H), δ 2.64 (t, *J* = 7.1 Hz, 1H), δ 2.58 (t, *J* = 7.0 Hz, 19H), δ 2.53 (d, overlapped, *J* = 7.7 Hz, 1H), δ 2.48–2.44 (m (t visual, overlapped), <1H), δ 1.89–1.77 (m, 20H), δ 1.19 (s, br, 27H).

#### APC(+/−)80/20 Synthesis

^1^H NMR (600 MHz, DMSO-*d*_6_ with TMS at a volume fraction of 0.03%, spectra referenced to 0.0 ppm): δ 5.87 (ddd, *J* = 15.5, 9.7, 4.6 Hz, 1H (set)), δ 5.28 (d, *J* = 17.3 Hz, 1H), δ 5.20 (d, *J* = 10.4 Hz, 1H), δ 4.60 (s, br, 1H (2H expected)), δ 4.31–4.18 (m, 17H), δ 4.15 (t, *J* = 6.1 Hz, 7H), δ 2.91 (s, br, 6H), δ 2.71 (s, br, 6H), δ 2.63 (t, *J* = 7.0 Hz, 1H), δ 2.57 (t, *J* = 6.6 Hz, 6H), δ 2.52 (d (overlapped), *J* = 7.1 Hz, 1H), δ 2.48–2.45 (m (t visual, overlapped), <1H), δ 1.87–1.76 (m, 7H), δ 1.19 (s, br, 10H).

#### APC(+/−)70/30 Synthesis

^1^H NMR (600 MHz, DMSO-*d*_6_ with TMS at a volume fraction of 0.03%, spectra referenced to 0.0 ppm): δ 5.92–5.82 (m, 1H (set)), δ 5.28 (d, *J* = 17.3 Hz, 1H), δ 5.20 (d, *J* = 10.2 Hz, 1H), δ 4.60 (s, br, 2H), δ 4.32–4.18 (m, 17H), δ 4.15 (t, *J* = 6.4 Hz, 6H), δ 2.94–2.88 (m, 5H), δ 2.73–2.66 (m, 6H), δ 2.63 (t, *J* = 6.9 Hz, 2H), δ 2.56 (t, *J* = 6.5 Hz, 5H), δ 2.52 (d (overlapped), *J* = 6.3 Hz, 1H), δ 2.47–2.44 (m, t visual, <1H), δ 1.87–1.77 (m, 7H), δ 1.18 (s, br, 9H).

#### APC(+/−)50/50 Synthesis

^1^H NMR (600 MHz, DMSO-*d*_6_, spectra referenced to 2.5 ppm): δ 8.54 (m, br, 5H), δ 5.87 (ddd, *J* = 15.1, 9.5, 4.2 Hz, 1H (set)), δ 5.28 (d, *J* = 18.1 Hz, 1H), δ 5.19 (d, *J* = 10.1 Hz, 1H), δ 4.60 (d, br, *J* = 3.3 Hz, 2H), δ 4.29–4.18 (m, 28H), δ 4.18–4.11 (m, 15H), δ 2.96–2.81 (m, 10H), δ 2.68 (s, br, overlapped, 6H), δ 2.63 (s, br, overlapped, 8H), δ 2.58–2.53 (m, 7H), δ 1.90–1.74 (m, 14), δ 1.18 (s, br, 18H).

### Polymer/Protein Complex Preparation

Polymer/protein complex mixtures were prepared using the following general procedure. The polymer at 5.0 mg/mL in PBS and protein at 5.0 mg/mL were prepared independently by dissolving the solid macromolecule with PBS dispensed from a micropipette. Usually, the polymer was massed by scraping the material out of one vial and smearing it to the inside of a new tared vial or microcentrifuge tube. Each solution was vortexed to ensure dissolution. Brief centrifugation using a benchtop centrifuge aided in recovering the liquid from foams that formed during mixing. Protein solutions were filtered using 0.1 μm polyvinylidene difluoride (PVDF) (Millipore, Millex, low protein binding) syringe filter units to remove dust and large aggregates, while polymer solutions were filtered with 0.22 μm PVDF syringe filter units. Both solutions were filtered directly into clean cuvettes for DLS. In this way, the solutions were diluted so that the polymer and protein concentrations were 2.5 mg/mL each in the final solutions, which were initially mixed by pipetting and then vortex mixing. The foam formed upon vortex mixing was allowed to settle prior to characterization by DLS.

### DLS and Zeta Potential

DLS and zeta potential measurements were made using a Malvern Zetasizer Nano ZS equipped with a 532 nm laser, a back-scattering detector (173°), and a forward-scattering detector (12.8°). In DLS, the autocorrelation functions were obtained with Malvern Zetasizer Software 7.13, and the size distributions were given by the inverse Laplace transformation of the autocorrelation functions, following the CONTIN algorithm (processed by the software automatically). Disposable polystyrene cuvettes and caps were flushed inside and out with copious amounts of filtered water and allowed to dry in a covered environment to prevent dust from settling on them. Protein and polymer solutions were typically prepared at 5.0 and 2.5 mg/mL, respectively, while complex solutions (protein/polymer mixtures) were characterized where each component was 2.5 mg/mL unless otherwise stated. Disposable, folded capillary zeta potential cells were used in zeta potential measurements. Automatic attenuation and voltage selection were used for all samples. Monomodal analysis was applied because of the high conductivity of the solution and yielded the mean particle mobility of the solution per run instead of a distribution. The zeta potential values were calculated using the Smoluchowski model provided by the same software as in DLS and averaged over three consecutive runs to give the standard deviations.

### Small-Angle Neutron Scattering

SANS measurements were performed on the VSANS instrument at the National Institute of Standards and Technology Center for Neutron Research. Cold neutrons with a wavelength (λ) of 6.0 Å and a spread (Δλ/λ) of 12% were used. The samples were prepared using 1× PBS prepared in deuterium oxide, loaded into 1 mm or 2 mm path length cylindrical quartz cells (Hellma), and mounted in a temperature-controlled sample environment. The scattered intensity, *I*(*q*), was measured as a function of scattering vector (*q*), where *q* = (4π/λ)sin(θ/2) and θ is the scattering angle. Data were taken using two detectors with fixed sample-to-detector distances of 1.1 and 5.1 m or of 4.6 and 18.6 m. The data were converted to an absolute scale using a direct measurement of the beam flux, the scattering volume, the sample transmission, the sample-to-detector distance, the detector efficiency, and the solid angle subtended by one detector pixel. Data reduction and analysis were performed in Igor Pro.^41^

### Analytical Ultracentrifugation

Analytical ultracentrifugation (AUC) was conducted in a Beckman-Coulter XL-I analytical ultracentrifuge using a Ti-50 rotor and 12 mm optical pathlength cells with sapphire windows. Measurements were performed at 20 °C after 3.5 h of pre-spin equilibration to ensure temperature homogeneity at 4400 rad/s. Radial absorbance scans were measured at 265 nm, as well as radial interference fringe shifts, with both at 4 min intervals. The density and viscosity of the PBS buffer and PBS plus 1.25 mg/mL polymer solution were measured separately in an Anton-Parr DMA 5000—LOVIS M densitometer–viscometer. The sample and reference volumes in each cell were 400 μL. Analysis of the recorded radial absorbance profiles as a function of time was conducted using the numerical fitting software SEDFIT (V16.1c) using the *c*(***s***) model.^42^ Parameters for the *c*(***s***) model were an *s*-value range of (0 to 12) sv (1 sv = 1 × 10^–13^ s) discretized with 241 values and a regularization of 0.95. The meniscus and noise were fit for each experiment but agreed well with the apparent positions in the data. A partial specific volume of 0.733 mL/g was used to analyze the bovine serum albumin (BSA) including samples. Friction factor values were floated to best fit values, with little deviation from the input estimate of 1.3. The sample concentrations were 1.25 mg/mL for BSA and APC(+/−)90/10 individually and 0.625 mg/mL BSA + 0.625 mg/mL APC(+/−)90/10 for the complex, with all in PBS buffer with pH = 7.4.

## Results and Discussion

### Synthesis and Molar Mass of the APC Precursor

An aliphatic polycarbonate backbone was selected for the polyampholyte design because of its biodegradability, which is relevant to its intended application as a potential protein carrier. The alkene moiety of the MAC monomer **1** enables post-polymerization modification to bear charged side chains that promotes the water solubility and protein binding. Our APC precursors were synthesized by the ring-opening polymerization (ROP) of MAC, catalyzed by an organo-urea catalyst developed by Lin and Waymouth.^39^ This urea anion catalyst **2** was selected based upon the proven selective and rapid polymerization with high yields and low polydispersity of a cyclic aliphatic carbonate similar to MAC. The solubility of **2**, as per the report, was beneficial to the polymerization of **1** in THF.^39^ The polymerization is clearly indicated by the convergence of the two proton signals α to the carbonate in MAC (labeled as C and C′ in Figure S1a). ROP resulted in a high monomer conversion percentage of 95% (Figure S2); the yield, however, was relatively low (63%) due to the polymer loss during the precipitation and dialysis steps, suggesting that the crude APC precursors may contain one-third of low-molar-mass species. The MAC monomer was not purified; therefore, the presence of 3% impurity cannot be ruled out with respect to a source of the lower yield and polydispersity. However, the target molar mass was close to the theoretical suggestive that monomer purity may not be the primary origin of the broad molar mass distribution.

**Figure 1:**
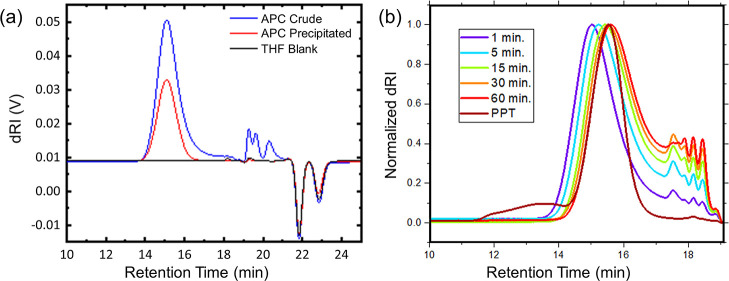
SEC traces of (a) crude and purified APC precursor and (b) reaction aliquots taken from a separate polymerization, showing the refractive index detector signals, and the precipitated (PPT) polymer. THF is used as the eluent in both cases.

SEC with light scattering detection characterized the absolute number-average molar mass (*M*_n_) and polydispersity (*D̵*) of both crude and purified APC precursors. The SEC trace of the crude product revealed a low-molar-mass tail, as opposed to just the single main peak in the purified one, indicating that short-chain impurities were removed by triplicate precipitation (Figure 1a). Notably, the amount of short chains significantly increases with the polymerization time beyond 1 min (Figure 1b). This is further supported by the relatively large *D̵* and the fact that *M*_n_ < 100·*M*_0_ (∼20,000 g/mol), where 100 is the molar ratio between the monomer and the initiator, and *M*_0_ is the molar mass of the monomer. The urea anion catalyst shows more selective ring opening with high rates of propagation; however, the relatively slower rate of chain-transfer side reactions such as transesterification leads to a broadening of the molar mass distribution, especially at high conversions when the monomer concentration is low.^39^ This appears to be observed in the present polymerization of **1** with molar mass distribution broadened as seen in SEC chromatograms due to the use of a highly active catalyst with chain transfer via transesterification. Empirically, such undesirable side reactions can be mitigated by decreasing the reaction time and temperature. Therefore, in the current study, we performed the polymerization below 0 °C for less than 20 s. Despite our attempt to control the reaction temperature and time, the effects of chain transfer by transesterification lead to larger than expected *D̵*. The target molar mass was based on the fixed monomer/initiator ratio (100/1) with an average degree of polymerization less than the theoretical (100) as calculated from the APC precursor absolute number-average molar mass and *M*_0_. Nevertheless, the critical pendant ene moieties remain intact during the polymerization, and the resulting APC precursors have the desirable molar mass, which suit our purpose of post-polymerization functionalization toward APC polyelectrolytes.

NMR may also be used to determine the degree of polymerization by end group analysis. However, in the present case, the end group signal was not unique. In retrospect, an alternate initiator, such as benzyl alcohol, would provide unique aromatic protons with a clearer end group signal but only in a case where impurities are eliminated that may also initiate. The CH_2_ α to the terminal OH group with validated peak assignment and signal would be more reliable. In this way, end group analysis could be used in conjunction with SEC.

### Synthesis and Side-Chain Characterization of APC Polyelectrolytes

**Figure 2:**
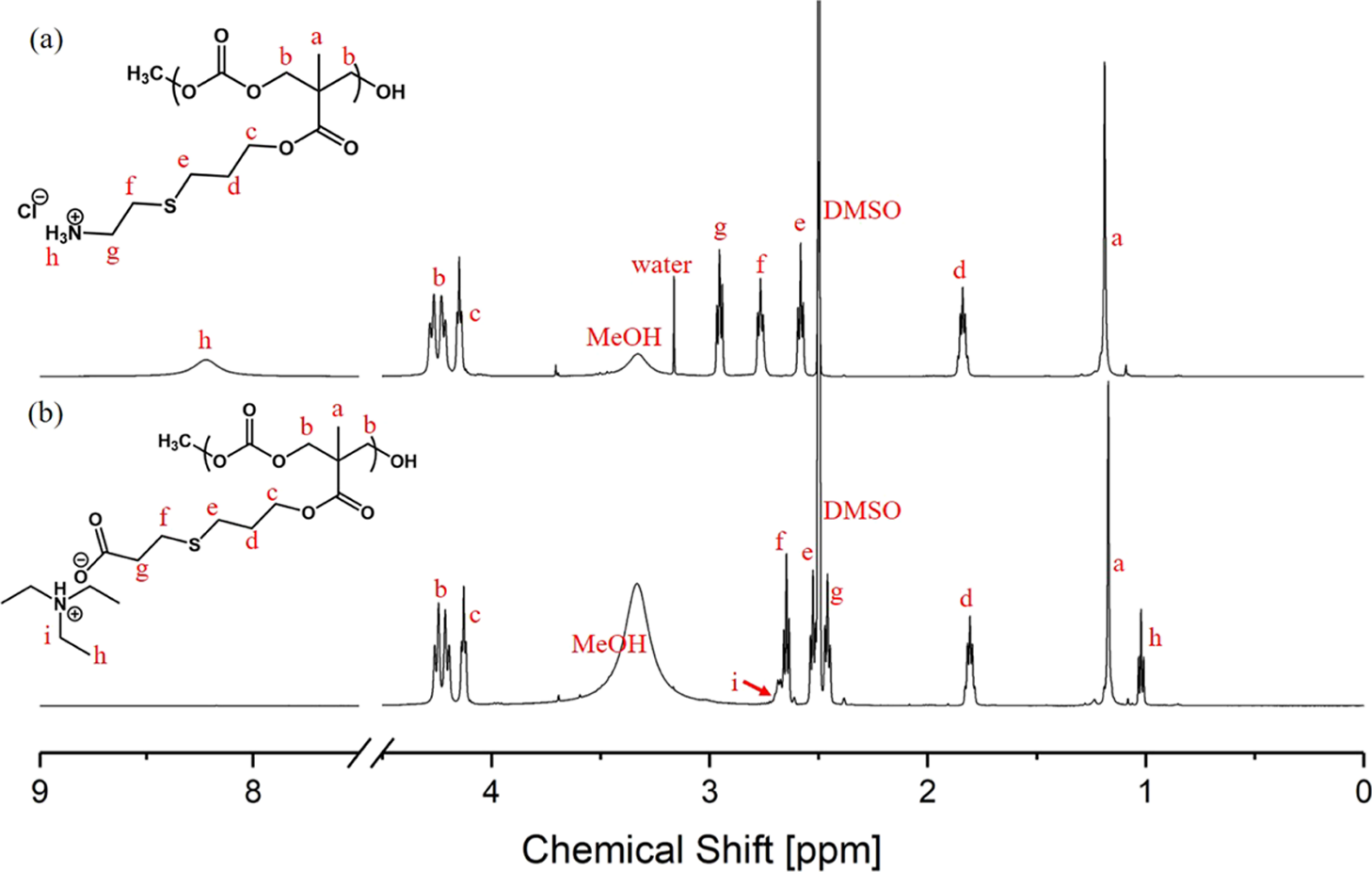
^1^H NMR spectra of (a) APC(+)100 and (b) APC(−)100 (in DMSO-*d*_6_, 600 MHz).

The polycation, APC(+)100, was prepared by directly conjugating cysteamine HCl to the APC precursor, following a literature protocol.^43^ The side-chain functionality of the dialyzed polymer was characterized by ^1^H NMR, which displayed a quantitative conversion from ene to ammonium thio ether. A new proton resonance, appearing as a quintet, was noted in the ^1^H NMR at ∼1.8 ppm representing the new methylene (−CH_2_−) unit that was formed as a result of the radical-mediated, thiol–ene reaction (Figure 2a). This resonance served as a diagnostic peak for monitoring the post-polymerization reaction. Similarly, the polyanion counterpart, APC(−)100, was also synthesized, following adaptations of previously reported procedures.^29^ To produce a PBS-soluble polyanionic APC, MPA was pre-mixed with TEA under ambient conditions. The mixture was vortexed to facilitate the acid–base reaction to produce a charged 3-mercaptopropionate with a triethylammonium counterion. The hydrophobic organic counterion was used since sodium or potassium 3-mercaptopropionate may not have had good solubility in the reaction solvent, THF. Protonated MPA was also conjugated to APC, yielding APC (COOH) 100 with carboxylic acid pendant groups; however, the protonated side chains rendered the polymer insoluble in water. In contrast, triethylammonium made both the starting thiol and the product polymer soluble in THF and water and therefore was the ideal counterion in this study. Again, quantitative conversion of the ene groups was indicated by ^1^H NMR, featuring the diagnostic quintet peak at ∼1.8 ppm (Figure 2b), as in the case of APC(+)100.

**Figure 3:**
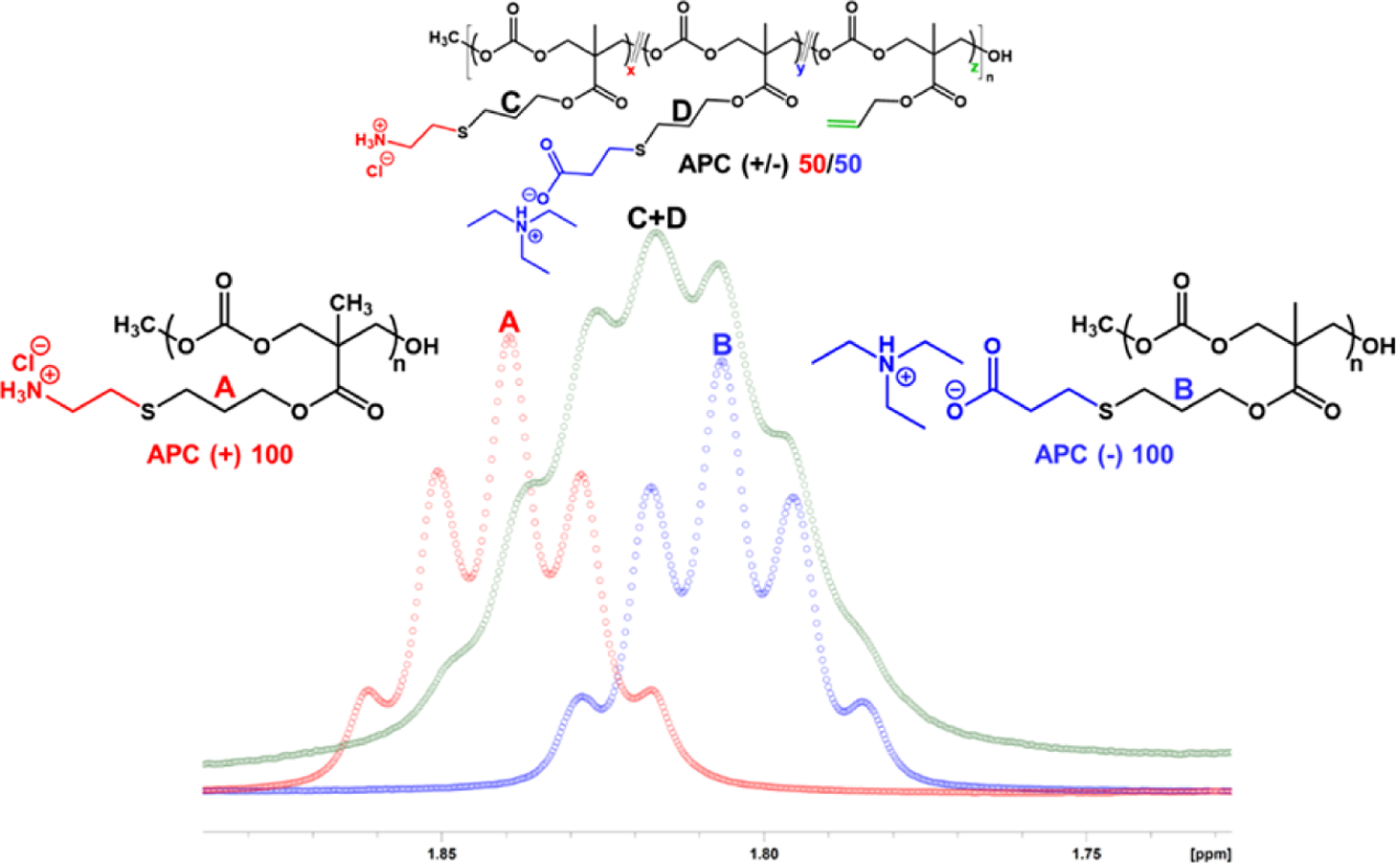
Overlaid ^1^H NMR spectra of dialyzed polymers (in DMSO-*d*_6_, 600 MHz): APC(+)100 in red, APC(−)100 in blue, and APC(+/−)50/50 in green, showing the signals of −CH_2_– protons β to the side-chain ester and thioether around 1.8 ppm. Peak intensities are scaled arbitrarily for clarity.

The synthesis of the homopolyelectrolytes confirmed the suitability of the precursor APCs for functionalization via thiol–ene chemistry. For the APC polyampholytes that contain both positively and negatively charged side chains, a one-pot reaction strategy was used, as in reaction (4) of Scheme 1. The result of this approach was a series of polyampholytes with statistical charge distributions and net charge dictated by the feed ratio between ammonium and carboxylate thiols. The initial conversion was low, as demonstrated by ^1^H NMR, due to the decreased solubility of the APC polyampholytes in THF, making the reaction partially heterogeneous; re-initiation of the reaction with a new photoinitiator (Irgacure 2959) and an increased reaction time were used to achieve higher conversion. The diagnostic, stand-alone quintet peak that was clearly resolved in the homopolyelectrolyte ^1^H NMR spectra became a multiplet for the polyampholytes with features derived from the overlap of each quintet from the respective cationic and anion side chains. This overlapped, complex multiplet was most clearly observed in the APC(+/−)50/50 spectrum (Figure 3). Saturating the DMSO-*d*_6_ solution of APC(+/−)50/50 with LiCl increased the resolution of the individual diagnostic multiplets in the ^1^H NMR spectrum (Figure S3d). It is worth noting that such multiplets were only used to determine the ene conversion in the reaction aliquots; the side-chain compositions of the final purified polymers were determined by a different set of peaks, that is, the unreacted ene groups (−C*H*=CH_2_−) at ∼5.9 ppm, the cationic −CH_2_– linker at ∼2.9 ppm, and the anionic −CH_2_– linker at ∼2.6 ppm (Figure S3).

Table 1 summarizes the key characteristics of polyelectrolytes and polyampholytes. The polyelectrolytes (entries 1–3) include a partially charged analogue, APC(+)93 bearing 7% ene monomer. Notably for the polyampholytes (entries 4–7), despite the unreacted ene groups (with the composition ranging from 5 to 20%), the final ratio of the cations and anions closely resembles the feed ratio, suggesting equal reactivity of the two thiol species. The observation of the decreased solubility of the polyampholytes in THF also led to a lower yield and an increased undesirable -ene content. We speculate that solubility was the source of the lack of quantitative conjugation in polyampholytes. The approach of tuning the side-chain functionality of polycarbonates by changing the starting feed ratio was sufficient for the purposes since the final and feed ratios are within 5% difference from each other. Therefore, the APC polyampholytes used in this study were named after the cation/anion ratio in the feed for simplicity. The absolute number-average molar mass provided in Table 1 refers to the APC precursors and not the post-polymerization modification polyelectrolytes.

**Table 1:** Characterization of Polyelectrolytes and Polyampholytes Prepared from Different Batches of the APC Precursor Polymer

	APC precursor polymer			post-polymerization side-chain composition^e^					
polymers^a^	*M*_n_ (kg/mol)^b^	*D̵*^c^	DP^d^	cation (%)	anion (%)	ene (%)	cation/anion	yield (%)	*R*_H_ (nm)^f^
APC(+)93	16.1	1.38	80	93	0	7	100/0	63	4.3 ± 1.8
APC(+)100	14.7	1.33	73	100	0	0	100/0	67	4.3 ± 1.9
APC(−)100	14.7	1.33	73	0	100	0	0/100	31	4.4 ± 1.8
APC(+/−)90/10	14.1	1.36	70	90	5	5	95/5	39	2.5 ± 1.3
APC(+/−)80/20	14.1	1.36	70	68	14	18	83/17	57	3.4 ± 1.6
APC(+/−)70/30	14.1	1.36	70	60	20	20	75/25	50	3.9 ± 2.2
APC(+/−)50/50	14.1	1.36	70	46	46	8	50/50	34	

a Molar feed ratio of cations/anions appears after the parenthesis.

b Refer to the absolute number-average molar mass of the APC precursors characterized by SEC.

c Dispersity of the APC precursors characterized by SEC.

d Degree of polymerization of the APC precursors characterized by SEC.

e Values determined by ^1^H NMR on post-polymerization modification of APC precursors into polyelectrolytes and polyampholytes.

f Pooled mean of hydrodynamic radii (*R*_H_) ± one pooled standard deviation from DLS.

Post-polymerization modification and -ene groups could potentially lead to cross-linking as a side reaction for the thiol–ene reaction, especially with difunctional thiols. The ^1^H NMR integral values of the methylene protons from the conjugated thiol linker and the backbone protons, labeled as f and b, respectively, in Figure 2 have a ratio of 1:2. Therefore, cross-linking as a side reaction appears below detection.

### Characterization of APC Polyelectrolytes and Polyampholytes in Aqueous Solution

Solution characterization confirmed the post-polymerization modifications of APCs. The solubility and phase behavior in PBS were studied since these are critical for applications as aqueous protein complexation agents. APC(+)100, APC(+)93, and APC(−)100 were soluble in PBS at pH = 7.4, indicating that the side-chain modifications imparted water solubility to the hydrophobic APC precursor. APC(+/−)90/10 and APC(+/−)80/20 were also soluble in PBS. On the contrary, although synthetically possible, APC(+/−)50/50 and an analogue, APC(+/COOH)50/50, were insoluble, while APC(+/−)70/30 had decreased solubility in PBS.

**Figure 4:**
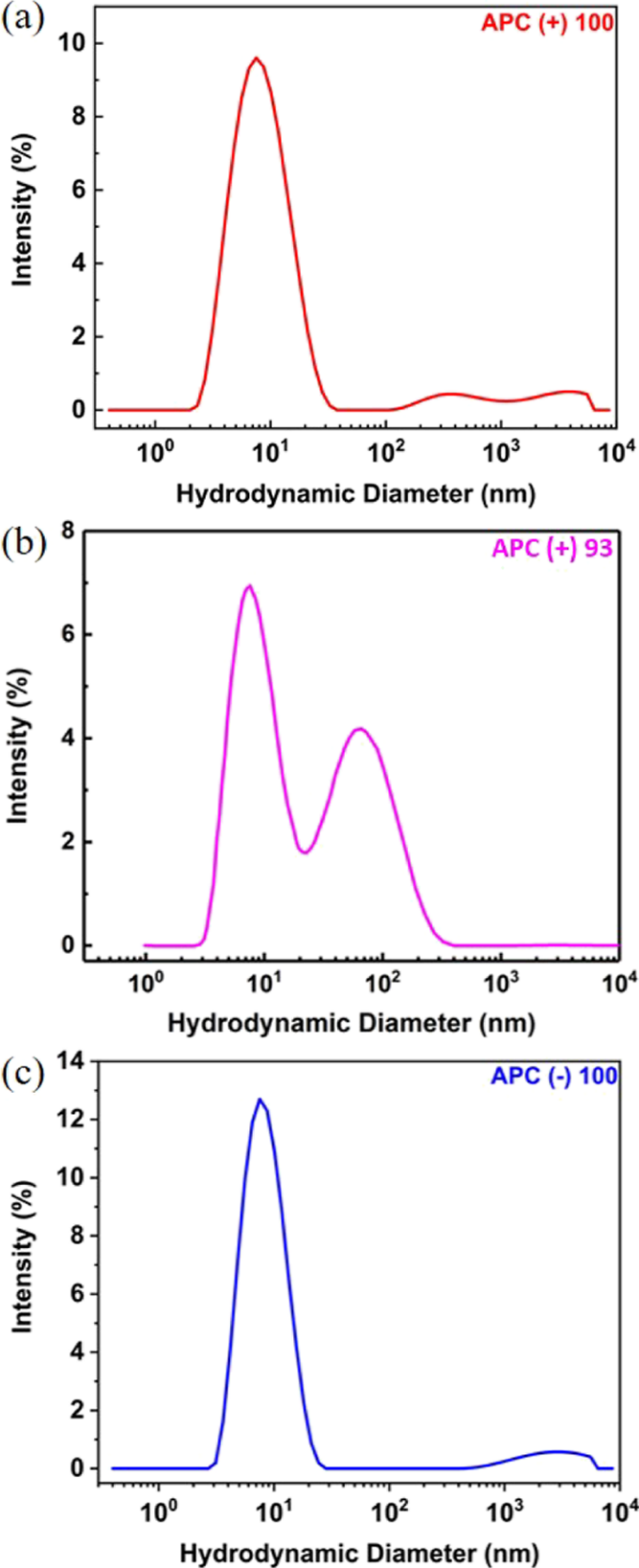
Representative hydrodynamic diameter (*D*_H_) distributions from DLS of (a) APC(+)100, (b) APC(+)93, and (c) APC(−)100, with all at 2.5 mg/mL in PBS, with pH = 7.4.

The series of polyelectrolytes was characterized by DLS for the hydrodynamic diameter (*D*_H_) distributions. As shown in Figure 4 and Table 2, APC(+)100 at 2.5 mg/mL exhibits one primary size distribution with average *D*_H_ = 8.6 nm, accompanied by additional lower amplitude modes of larger *D*_H_. Its analogue APC(+)93 also has a primary peak at *D*_H_ = 8.6 nm, albeit with more significant amount of larger species. Both polycations aggregate over time in solution, which is evident in the time-resolved DLS (Figures S4 and S5), but this effect is more pronounced in APC(+)93, presumably due to the association of the ene groups. For the polyanion, APC(−)100 has similar *D*_H_ distribution to APC(+)100, again indicating that the additional hydrophobicity introduced by the unreacted side chains is likely the cause of the polymer aggregation. The zeta potentials (Table 2) are −14.4 mV for APC(−)100, +20.0 mV for APC(+)93, and +9.90 mV for APC(+)100, which qualitatively agree with the expected sign for polyanions and polycations.

**Table 2:** Characteristics of the APC Polyelectrolyte/Protein Complexes

	*R*_g_ (nm)	*R*_H_ (nm)^c^	*R*_g_/*R*_H_	zeta potential (mV)	semimajor axis, *R*_A_ (Å)^d^	semiminor axis, *R*_B_ (Å)^d^
BSA	3.08 ± 0.03^b^	4.5 ± 1.5	0.68 ± 0.23		60.3 ± 0.7	23.5 ± 0.2
lysozyme		2.3 ± 0.9				
APC(+)93	3.28 ± 0.14^a^	4.3 ± 1.8	0.77 ± 0.32	20.0 ± 1.6		
APC(+)100	2.63 ± 0.07^a^	4.3 ± 1.9	0.60 ± 0.27	9.9 ± 3.8		
APC(+)93 w/BSA	4.15 ± 0.04^b^	6.1 ± 2.2	0.68 ± 0.25	6.2 ± 0.5	86.5 ± 0.9	23.9 ± 0.1
APC(+)100 w/BSA	4.03 ± 0.03^b^	5.5 ± 1.9	0.73 ± 0.25	5.2 ± 0.3	83.8 ± 0.8	23.5 ± 0.1
APC(−)100 w/lysozyme		3600 ± 700				

a Determined by SANS in PBS-D_2_O (Figure S11 and Table S2).

b Calculated by *R*_g_ = √(1/5) (*R*_A_^2^ + 2*R*_B_^2^).

c Determined by DLS.

d Determined by fitting the SANS data in Figure 8 to the ellipsoid models.

**Figure 5:**
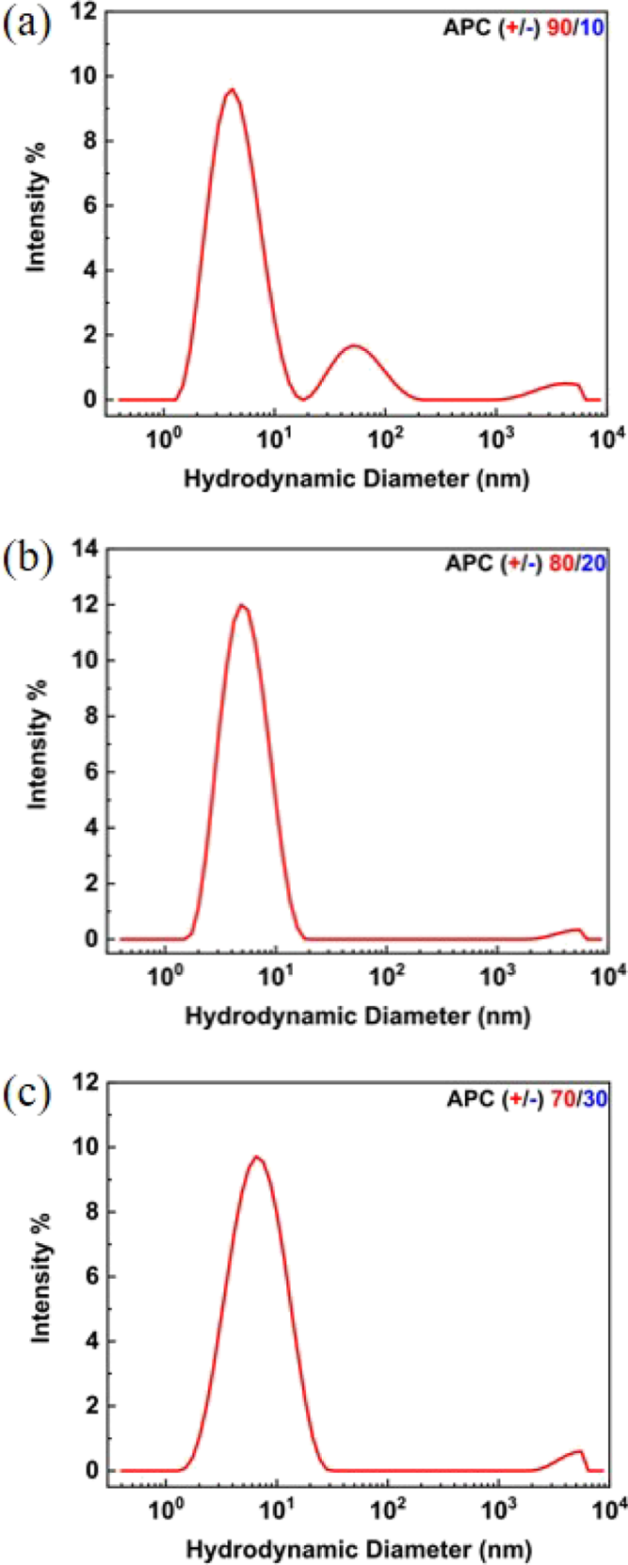
Representative hydrodynamic diameter (*D*_H_) size distributions from DLS of (a) APC(+/−)90/10, (b) APC(+/−)80/20, and (c) APC(+/−)70/30, with all at 2.5 mg/mL in PBS with pH = 7.4.

For the polyampholyte solutions, as demonstrated in Figure 5, APC(+/−)90/10 has a multimodal size distribution with a primary peak at *D*_H_ = 5.0 nm, while APC(+/−)80/20 and 70/30 are nearly monomodal, with primary peaks at 6.8 and 7.8 nm, respectively. It is worth noting that APC(+)100 and APC(+/−)90/10, 80/20, and 70/30 have similar degrees of polymerization based upon the precursor APC characterization; therefore, their chain dimensions under identical conditions should follow (+)100 > (+/−)90/10 > (+/−)80/20 > (+/−)70/30 theoretically since the net electrostatic interactions favor attraction more as charge neutrality is approached.^44,45^ Nevertheless, this is not the case in our observation, at least partially owing to the complexity of chain aggregation. The ionic side chains may serve to catalyze the degradation by methanol during the dialysis step and lead to a change in degree of polymerization. The smaller *D*_H_ for APC(+/−)90/10 may be affected by a lower average degree of polymerization after post-polymerization modification and subsequent dialysis. However, we would expect this to occur to all polymers, which was not the case due to their relative sizes by DLS. As a test of this mechanism, aqueous SEC analysis compared precipitated versus precipitated and dialyzed polymers and did not show a substantial change in molar mass (Figure S14) between APC(+)93 and APC(+)100.

Compared with APC (+/−) 90/10 and 80/20, APC(+/−)70/30 has only marginal solubility in PBS buffer, suggesting self-coacervation. At charge neutrality, that is, APC(+/−)50/50, the polymer is insoluble (phase-separated), consistent with previous theories and experiments.^45−48^^1^H NMR as independent evidence supports the self-coacervation hypothesis as APC(+/−)50/50 in DMSO-*d*_6_ displays broad and poorly resolved peaks in the absence of a salt, in contrast to the more clearly resolved peaks in the LiCl-saturated solvent (Figure 3). Self-association and molecular aggregation are known to broaden peaks in solution NMR;^49^ therefore, the improvement in the peak resolution suggests that LiCl dissociates the previously self-coacervated polymer.

**Figure 6:**
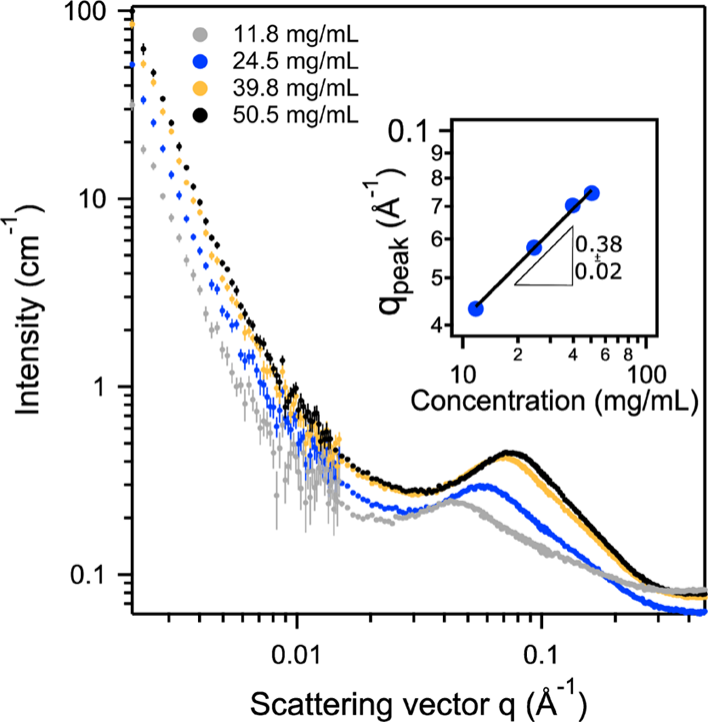
SANS of APC(+)93 in D_2_O at various concentrations. The inset figure shows the power law scaling of *q*_peak_ with polymer concentration.

SANS was used to study the structure of selected APC polyelectrolytes and polyampholytes in low-ionic-strength solutions. As shown in Figure 6, two main features were observed for the polycation APC(+)93. The significant increase of scattering intensity at low *q* and a correlation peak at intermediate *q* were observed. The former is characteristic of large domains formed by associating chains, which is consistent with the multimodal DLS size distribution (Figure 4b). The latter indicates a concentration-dependent correlation length between highly charged polyelectrolytes.^50^ As the polycation concentration increases from 11.8 to 50.5 mg/mL, the peak scattering vector, *q*_peak_, scales with *c*^0.38 ± 0.02^. The scaling for highly charged polyelectrolytes is typically *q*_peak_ ∼ *c*^1/3^ when *c* < *c*_κ_ and *q*_peak_ ∼ *c*^1/2^ when *c*_κ_ < *c* < c*. The overlap concentration c* is well known as the concentration above which the polymer chains start to interpenetrate, where c* = 3*M*/4π*N*_A_*R*_g_^3^. The critical electrostatic correlation concentration *c*_κ_ is defined as the concentration above which the polymer chains start to feel the electrostatic interaction with their nearest neighbors, and similarly, *c*_κ_ = 3*M*/4π*N*_A_(κ^–1^)^3^ (*N*_A_ and κ^–1^ are the Avogadro number and the Debye screening length, respectively). For the polycation APC(+)93, we use *M*_n_ = 24,000 g/mol estimated based on the APC precursor molar mass and the NMR calculated molar ratio of side chains, and *R*_g_ ≈ *R*_h_ ≈ 4 nm estimated by DLS for the calculation. This gives c* = 130 mg/mL, and therefore, *c* < c* is always valid. On the other hand, under salt-free conditions (the polymer in pure D_2_O), *c*_κ_ should be vanishingly small. Therefore, the observed *q*_peak_ scaling is closer to dilute regime (*c*_κ_ < *c* < c*) limiting law of *c*^1/3^ rather than the expected result of *c*^1/2^ for semidilute solutions.^51,52^ Deviations from the semidilute scaling were observed.^53^

Overall, the APC polyelectrolytes and polyampholytes were characterized by DLS, zeta potential, and SANS measurements, and the results are consistent. Although aggregation is prevalent in these polymer solutions, the primary species is the solvated single polymer chains in the buffered solutions as indicated by both DLS and SANS (Supporting Information).^54,55^

### Characterization of APC Polyelectrolyte/Protein Complexes

**Figure 7:**
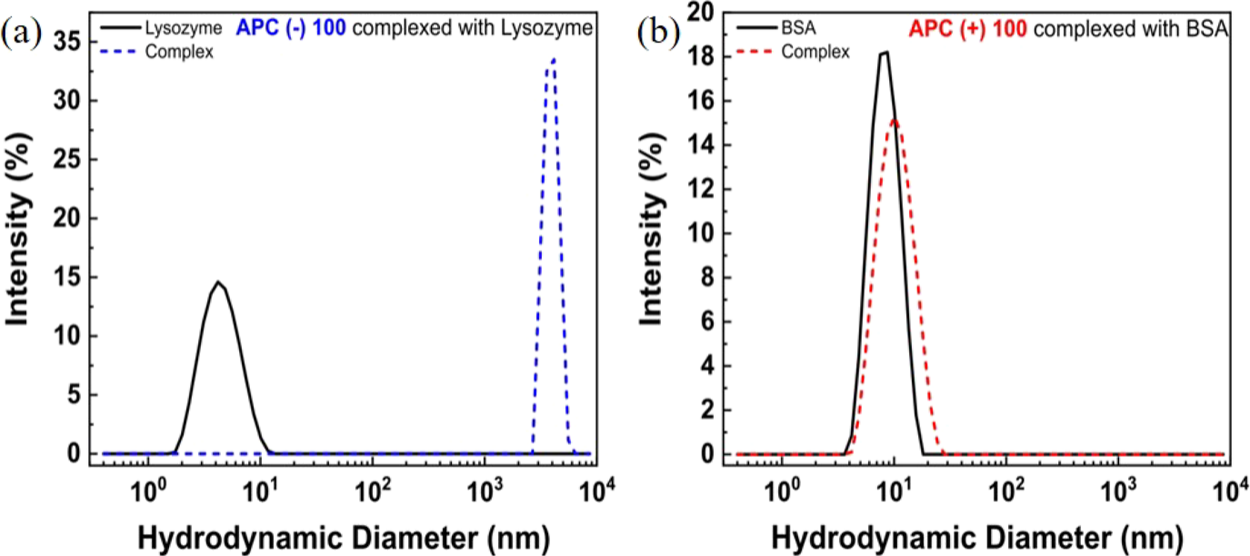
Hydrodynamic diameter (*D*_H_) distributions of proteins (solid lines) and complexes (dashed lines) formed by (a) APC(−)100 + lysozyme and (b) APC(+)100 + BSA. The solvent is the PBS buffer at pH = 7.4.

Model proteins, BSA and lysozyme C, were used to study the complexation behaviors of APC polyelectrolytes and polyampholytes. The isoelectric points (pI) of BSA and lysozyme are 4.7 and 11.0, respectively; therefore, BSA is negatively charged, and lysozyme C is positively charged at neutral pH (7.4).^56,57^ According to the charge complementarity principle, BSA preferentially binds with APC(+)100 and 93, while lysozyme should be more effective in complexing with APC(−)100. Figure 7 shows the size distributions of each protein, overlaid with complexes with APC polyelectrolytes. The *D*_H_ of free lysozyme and BSA are 4 and 8 nm, respectively, which agree with the reported literature values.^58−62^ Conversely, the *D*_H_ of the polyelectrolyte/protein complexes differ substantially from case to case. In Figure 7a, large complexes (*D*_H_ ∼ 3600 nm) form instantaneously after the mixing of APC(−)100 and lysozyme, leading to partial precipitation of the complex solution. Nevertheless, these complexes can be re-dispersed by prolonged vortex mixing with the stability, on the order of the measurements times. Ultimately, the solution is unstable and phase-separates (see Figure S6 in the Supporting Information for detailed information).

In stark contrast to APC(−)100/lysozyme, the complexation between the polycations and BSA results in translucent initial solutions that became completely clear after vortex mixing or after sitting unattended for more than 8 h, which indicates an equilibration process separating the initial kinetic state from the final thermodynamic state (Figure S7). The structure of the clear complex is shown in Figure 7b. While the two size distributions in Figure 7b are close to each other, there is a reproducible shift in hydrodynamic diameter peak position for the complexes (*D*_H_ = 11 to 12 nm), compared with the protein control (*D*_H_ = 8 nm), as summarized in Table 2. APC(+)93 has similar complexation behavior to APC(+)100, albeit with higher initial turbidity, owing to the chain association (Figure S8).

**Figure 8:**
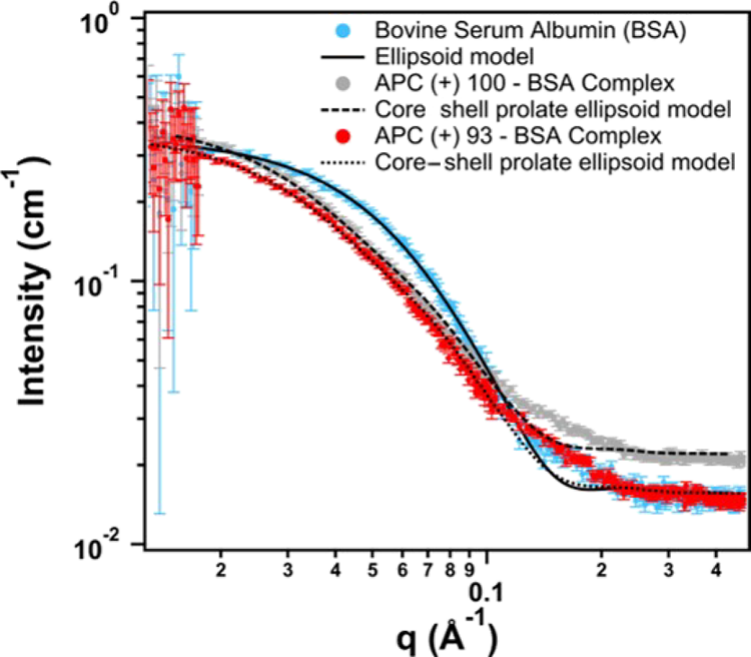
SANS of free BSA (cyan symbols), APC(+)93 + BSA (red symbols), and APC(+)100 + BSA (gray symbols) in PBS-D_2_O. The solid line is best fit to the ellipsoid model, and the dashed and dotted lines are best fit to the core–shell ellipsoid model.

In order to corroborate that BSA binds with the polycations, SANS was conducted on the protein and complexes prepared in PBS-buffered D_2_O. Figure 8 shows the scattered intensity in the intermediate *q* region featuring the complexes and the bare protein. The data of BSA were fit to the ellipsoid (prolate) model consistent with the work by Bendedouch and Chen.^63^ In this case, the semimajor and semiminor axis are provided in Table 2 along with the calculated radius of gyration. Interestingly, the complexes of BSA with APC(+)100 (gray) or APC(+) 93 (red) exhibit a shift to lower *q* when compared to the protein (cyan). This is direct evidence of an increase in the radius of gyration (*R*_g_) of the BSA/polycation complexes relative to free BSA. In order to quantify this change in dimension, the prolate ellipsoid model of BSA was adjusted to a core–shell ellipsoid (prolate) model^64^ assuming BSA as the core and the polycations as the shell (Figure S13). As summarized in Table 2, the semimajor axis significantly increases after complexation with polycations, which unambiguously confirms the binding. However, the semiminor axis remains nearly constant, suggesting that the binding predominantly takes place at the two lengthened poles of the BSA prolate. A similar conclusion was found in the study of mixtures of surfactants and BSA.^65^ The models used are available within the Igor Pro SANS analysis packages and discussed in the Supporting Information.^41^ The calculated shape factors (*R*_g_/*R*_h_) are shown in Table 2 and are between 0.68 and 0.73 for the uncomplexed and complexed BSA samples in agreement within uncertainties to 0.775 for a compact object.

Despite the success in DLS and SANS characterization of the polyelectrolyte/protein complexes, there is still one fundamental question: why do the two similar model systems, polyanion/lysozyme and polycation/BSA, lead to disparate complexation results? Based on our calculation, the moles of net charges per gram of macromolecules are +0.62, −2.46, −0.26, and +3.19 mol/kg for lysozyme, APC(−)100, BSA, and APC(+)100, respectively (Table S3). Since the mass concentrations of polymers and proteins are identical (2.5 mg/mL), the calculated values above represent the charge ratios in each complex solution. As can be seen in the case of the polyanion/lysozyme, the polymer charges are in 4-fold excess of the protein’s complementary charges; in the polycation/BSA, this excess is more than 10-fold. Such a charge density imbalance leads to the charge reversal of BSA after complexing with polycations (Table 2).^66,67^ We speculate that the higher charge density of lysozyme (+0.62, compared with −0.26 for BSA) is the cause of macroscopic phase separation, although the charge ratio in the complex is far from stoichiometric. Our speculation is supported by the experimental results by Andrianov et al. that increasing the protein/polymer molar ratio of their highly charged polyphosphazene/avidin complexes, that is, moving toward charge stoichiometry, led to the formation of an insoluble precipitate and the study by Burova et al. with polyphosphazene/lysozyme.^68,69^ Similarly, Dubin and co-workers have also determined that there are distinct conditions where soluble complexes are favored over aggregates of complexes that phase-separate; these phase regions were identified by altering pH which changed the ionization states of charges on the protein surface in relation to its polycation partner.^10^

Along these lines, the adsorption of linear polyelectrolytes to uniformly charged spheres has been studied theoretically.^100^ A critical adsorption line can be determined based upon several molecular parameters including the polymer linear charge density, sphere radius, surface charge density, and Debye screening length. At a fixed polyelectrolyte size and sphere radius, a higher charge density is required for adsorption in the presence of a high salt. The PBS conditions used here are at quite a high salt concentration (considering the 1:1 electrolytes, NaCl and KCl, at a concentration of 0.1397 mol/L) such that the Debye screening length is 0.8 nm. This is a highly screened solution, yet soluble complexes are observed in the case of BSA while large clusters in the case of lysozyme. The observations of polyelectrolyte complexing to the semimajor axis in BSA are illustrative of the additional importance of the local charge heterogeneity, rather than uniform charge. Lysozyme similarly exhibits an inhomogeneous charged surface such that additional short-range attraction was needed to observe polyelectrolyte binding by Monte Carlosimulation.^70^ A related study with polysaccharides binding to lysozyme exhibits large aggregates that are highly dependent upon an asymmetric mixing ratio at low ionic strength.^71^ Such an effect of asymmetric mixing ratio and association driven by the hydrophobicity of the proteins appear more relevant to explain our observations in the case of a high salt. This would fall into the case of soluble complexes versus coacervation.

### Characterization of APC Polyampholyte/Protein Complexes

**Figure 9:**
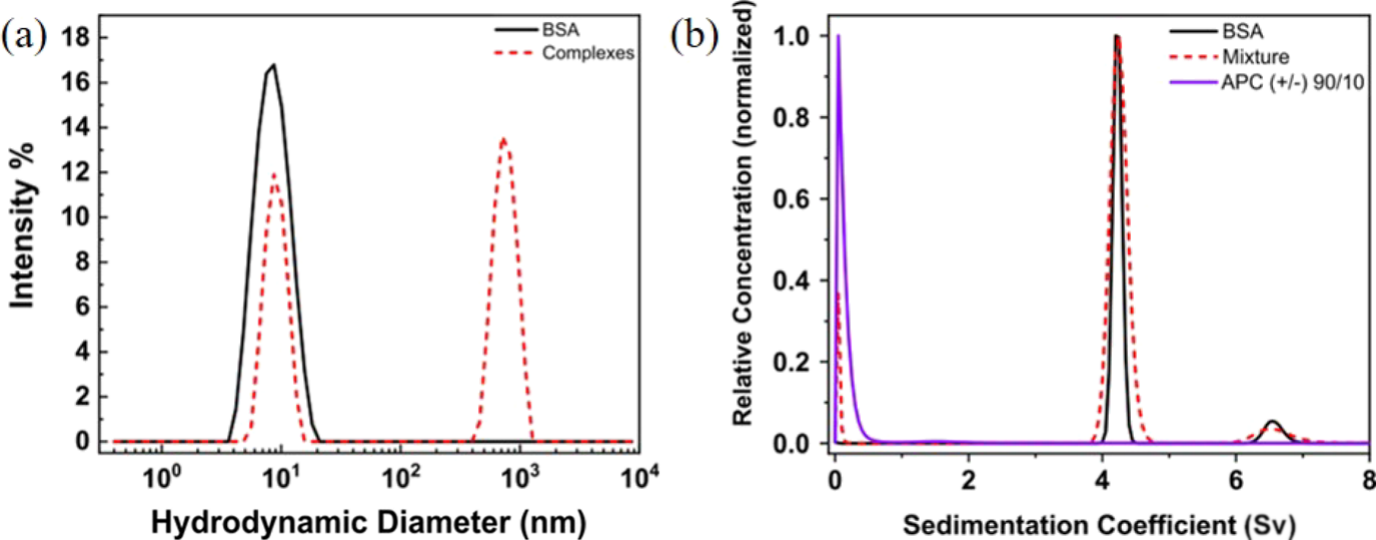
(a) Hydrodynamic diameter (*D*_H_) distributions of protein (solid lines) and the complex (dashed lines) formed by APC(+/−)90/10 and BSA. (b) Sedimentation coefficient distribution of the APC(+/−)90/10 + BSA complex (0.625 mg/mL each, red dashed line), overlaid with free BSA (1.25 mg/mL, black solid line) and APC(+/−)90/10 (1.25 mg/mL, violet solid line). The spin rate is at 42 krpm.

Since APC(−)100 produced insoluble precipitates when complexed with lysozyme, the anionic net charge end of the polyampholyte spectrum was not considered further; APC(+/−)50/50 was also excluded due to its poor solubility in PBS buffer. The other polyampholytes, APC(+/−)90/10, 80/20, and 70/30, are promising candidates for protein binding and delivery. APC(+/−)90/10 demonstrates unique behavior: upon mixing its solution with BSA, the resulting complex solution appeared clear relative to the other polymer/BSA complexes (Figure S9). DLS of these complexes revealed a bimodal size distribution with a large species at *D*_H_ = 680 ± 160 nm and a small species at *D*_H_ = 8.8 ± 2.0 nm, with the latter having almost the same hydrodynamic diameter as the native protein (Figure 9a).

In this case, AUC was used to validate the identity of the small species. As can be seen in Figure 9b, the two control experiments on pure APC(+/−)90/10 and BSA indicate that the former has a sedimentation coefficient of 0, which means that the polyampholyte does not sediment at the condition applied; the latter displays a bimodal distribution with a primary peak at 4 sv (BSA) and a secondary peak at 6 sv (BSA dimer, in a very small amount). The complex has the same sedimentation coefficient distribution as free BSA; therefore, we can conclude that the small species in Figure 9a is indeed the unbound protein. The difference in peak widths in the AUC result is likely due to the difference in BSA concentration of the two samples. The large species in DLS is not observed in the sedimentation coefficient distribution because the large complexes sediment too fast (>8 sv) under the experimental conditions beyond the limits of detection. We conclude that APC(+/−)90/10 complexes with BSA in the form of multiprotein and polyelectrolyte clusters that are larger than the polymer in PBS (Figure 5a). This behavior contrasts the well-defined complexes between BSA and APC(+)100 by DLS (Figure 7b) and validated by SANS (Figure 8). The lack of large clusters by DLS (Figure 7b) points to the sensitivity by which DLS is able to measure challenging polydisperse and wide-range-in-size-scale structures.

The incorporation of anionic side chains modifies the complexation behavior of APC polyelectrolytes on both macroscopic and molecular levels. Macroscopically, it was unexpected that the complexation between APC(+/−)90/10 and BSA produces initially a clear solution, whereas the polycations APC(+)100 and APC(+)93 do not. Moreover, APC(+/−)80/20 and APC(+/−)70/30 reproduce the behavior of the polycations, and their solutions turn cloudy upon complexation with BSA (Figure S10). Molecularly, the ∼5% anionic side groups fundamentally change the binding behavior and strongly favor large multi-BSA + multichain complexes. The role of unreacted ene groups should not be neglected, but these dilute solution studies show that binding of the net cationic APC(+/−)90/10 to the net anionic BSA favors large clusters. It is conceivable that this structure is stoichiometric-limited such that the APC(+/−)90/10 complex to the heterogeneously charged BSA leads to charge reversal and further clustering, then leaving behind uncomplexed BSA in solution. This speculation could be studied through systematic variation of the ratio of protein and the polyelectrolyte. The large aggregation may be further enabled by the presence of hydrophobic ene groups which distinguishes from the binding with APC(+)100.

## Conclusions

Rapid, organo-catalytic ROP of a commercial carbonate monomer was combined with post-polymerization modification using thiol–ene chemistry to synthesize a series of polyelectrolytes. The conjugation of ion-containing side chains to aliphatic polycarbonate backbones yielded highly charged polyelectrolytes, while polyampholytes with tunable net charge were prepared by adjusting the initial feed ratio of mixtures of thiol salts used in the post-polymerization scheme.

The solubility and solution behaviors of the APC polyelectrolytes and polyampholytes were studied with the combined efforts of DLS, SANS, and zeta potential. All the synthesized polymers displayed some degree of chain association in both water and PBS buffer except the one with 50/50 cation/anion side chains, which was insoluble as a result of self-coacervation. This self-coacervate effect could be observed by NMR via peak width resolution as a function of the added LiCl salt.

The complexation of APC polyelectrolytes was investigated using two model systems, polycations/BSA and polyanions/lysozyme, which led to striking differences: solutions of polycation/BSA complexes started out turbid and became clear, while solutions of polyanion/lysozyme complexes started out turbid and precipitated. Polymers with 100% cationic side chain composition preferably formed small soluble complexes with the net anionic charged BSA. Incorporating ∼5% of anionic side chains into otherwise cationic polymers was enough to deactivate the formation of small complexes with BSA and form large aggregates. This effect goes beyond polyelectrolyte complexation criteria to proteins based upon uniformly charged proteins but illustrates the role of charge heterogeneity of the protein and chain conformation of the polyampholyte. This was most convincingly shown by SANS results that the polycation was bound primarily to poles of the prolate ellipsoidal BSA. A further increase in the anionic side chain content also impacted macrophase solution behavior and affected the response of the polymer/protein mixtures. These experiments underscore the complexity of polymer–protein interactions and reinforced the notion that it is difficult to predict such complexation behavior a priori.

## Supporting Information Available

Additional experimental details and notes on polyampholyte syntheses and purifications; additional ^1^H NMR spectra for the commercial MAC monomer, crude polymerization aliquots, and purified polymers; time-resolved DLS of APC polyelectrolyte solutions; photographs of APC/protein complexes; SANS of polycations and the determination of their *R*_g_ in PBS-D_2_O; SANS core–shell ellipsoid scattering model; and values used for the calculation of charge ratios in the APC/protein complexes (PDF)

## Notes

Certain commercial equipment and materials are identified in this paper in order to specify adequately the necessary experimental procedures. In no case does such identification imply a recommendation by the National Institute of Standards and Technology (NIST), nor does it imply that the material or equipment identified is necessarily the best available for this purpose.
