# Supplementary material for: Tuning Net Charge in Aliphatic Polycarbonates Alters Solubility and Protein Complexation Behavior
Source: ACS Omega. 2021 Aug 26;6(35):22589–602. doi: 10.1021/acsomega.1c02523 (PMC8427630; doi:10.1021/acsomega.1c02523)
Supplement: Supplementary file 1 — ao1c02523_si_001.pdf [file ao1c02523_si_001.pdf]

SUPPORTING INFORMATION

Tuning Net-charge in Aliphatic Polycarbonates Alters Solubility and Protein Complexation Behavior

Nicholas D. Posey[1], Yuanchi Ma, Michael Lueckheide, Julia Danischewski, Jeffrey A. Fagan, Vivek M. Prabhu[*]

Materials Science and Engineering Division, Material Measurement Laboratory, National Institute of Standards and Technology[§], 100 Bureau Drive, Gaithersburg, Maryland 20899, USA

[‡]Official contribution of the National Institute of Standards and Technology; not subject to copyright in the United States of America

*Corresponding author: vprabhu@nist.gov

[1] Present address: Cambridge Polymer Group, Boston MA 02129

1H NMR characterization of APC Precursor and Small Molecule

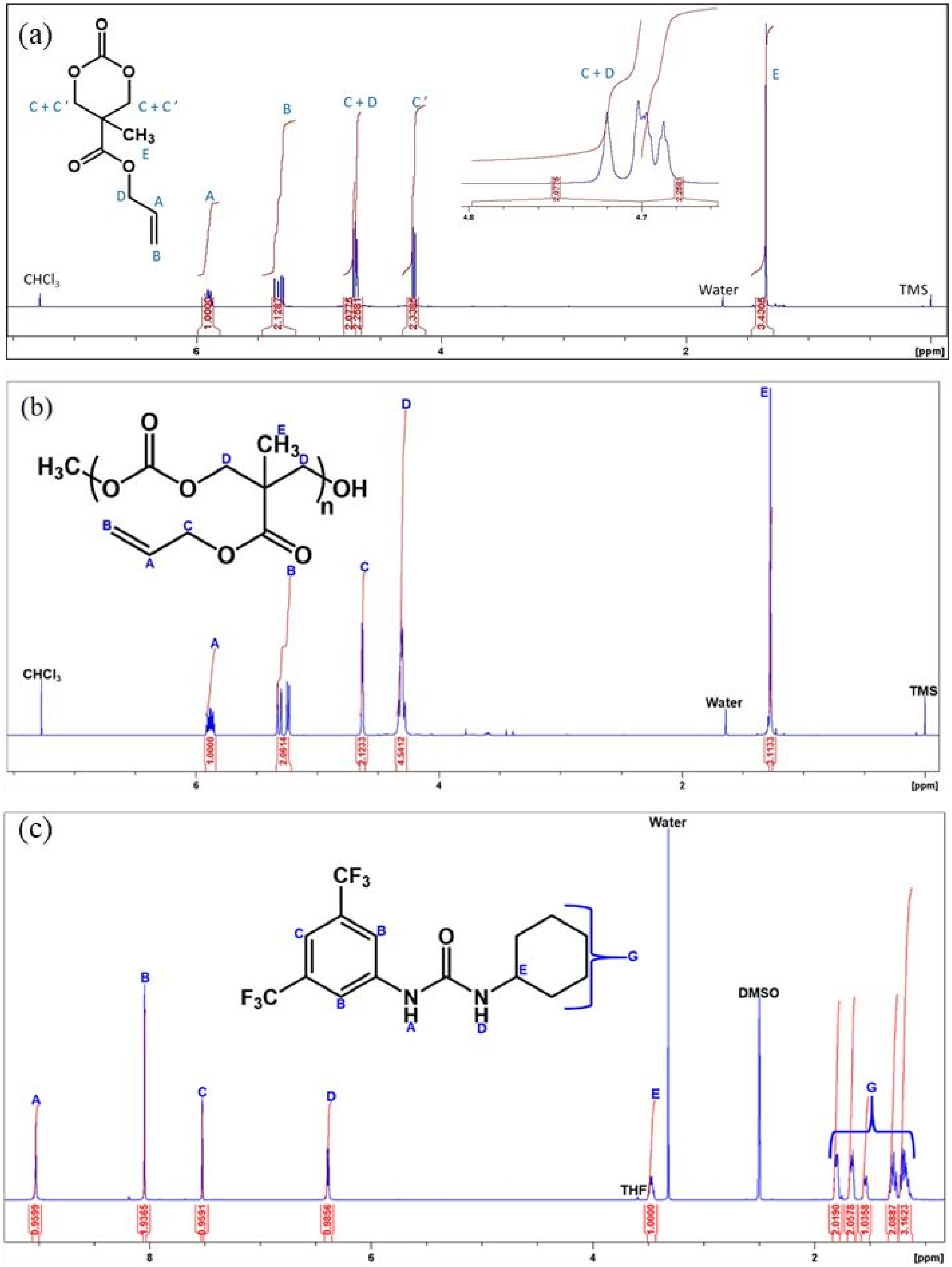

Figure S1. ^{1}H NMR (600 MHz, $CDCl_3$ with TMS) spectrum (produced in Top Spin) of the (a) commercial monomer 5-methyl-5-allyloxycarbonlyl-1,3-dioxan-2-one known as MAC and (b) APC precursor. (c) ^{1}H NMR (600 MHz, DMSO-d_6) spectrum (produced in Top Spin) of organo-urea catalyst.

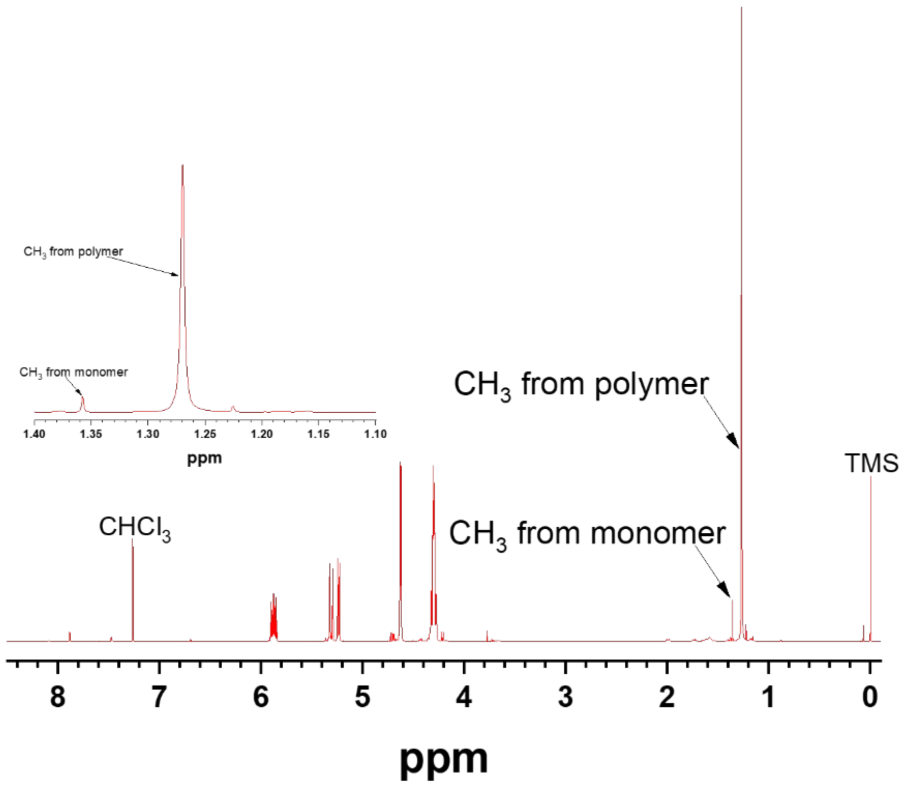

Figure S2. ^{1}H-NMR spectrum of the crude aliquot of the APC precursor synthesis, showing residual monomer relative to the polymer (600 MHz, in $CDCl_3$ with TMS). The $-CH_3$ protons of the monomer and the polymer (peaks E in Figure S1) were integrated to determine the degree of conversion as 96.4%.

Syntheses and Purifications of APC Polyampholytes

APC polyampholytes, APC (+/-) 90/10, 80/20, 70/30 and 50/50 were synthesized following the same general procedures outlined in the "Synthesis" section of the main text, while the specific modifications are summarized in Table S1.

Dialysis purification of APC(+)100

For purification by dialysis, regenerated cellulose membranes with molar mass cutoff of 3,500 kDa (Spectrum) were first equilibrated in methanol and then loaded with filtered polymer solution (0.45 µm PTFE syringe filter) dissolved in MeOH. The dialysis bath was a 1.5 L beaker filled with methanol. The first methanol bath was exchanged the next morning after equilibrating overnight, the second bath was exchanged at the end of the day, and the third methanol bath equilibrated again over night. The dialysis bag was removed, and the polymer was dried under high vacuum. To fully dry the polymer after the yield check, the polymer was freeze/thaw cycled in liquid nitrogen while under high vacuum. This caused the polymer to flake off the sides of the vial. ^{1}H NMR indicated that the polymer sample had a methanol mol fraction of 11.1 % and a polymer repeat unit mol fraction of 88.9 %, despite days of drying under high vacuum at room temperature.

Dialysis purification of polyampholytes

The dialysis procedure for the polyampholyte was different. The dialysis membrane was first hydrated and equilibrated in water, and then filled with polymer dissolved in methanol. A 250 mL graduated cylinder, filled with methanol, served as the dialysis bath. The first 250 mL bath was discarded after 30 min, and the dialysis bag and graduated cylinder were rinsed with methanol. The bag was put into a second ≈ 250 mL methanol bath. After ≈ 80 min the second methanol bath was discarded, the bag was rinsed with methanol, and then placed in a third ≈ 250 mL methanol bath which was equilibrated overnight resulting in the swelling of the bag. The third bath was discarded and replaced with a fourth ≈ 250 mL bath. The bag remained in the fourth bath for 2 h to 3 h at which time the fourth bath was discarded and replaced with a fifth and final ≈ 250 mL methanol bath. The polymer was removed from the dialysis bag, concentrated by rotary evaporation at room temperature, and transferred to a tared vial. In total, five ≈ 250 mL methanol baths were used in dialysis over a ≈ 22.5 h period where the first bath was discarded in less than 1 h and the fourth bath was equilibrated overnight.

Table S1. The reaction, precipitation and dialysis conditions of APC polyampholytes

Polyampholyte	Reaction solvent [a,b]	Precipitation solvent [a]	Dialysis solvent [a]
APC (+/-) 90/10	THF/MeOH	EtOH/Et_2O = 9/1	MeOH
APC (+/-) 80/20	THF/MeOH	EtOH/Et_2O = 8/2	MeOH
APC (+/-) 70/30	THF/MeOH	EtOH/Et_2O = 7/3	MeOH
APC (+/-) 50/50	THF/MeOH	EtOH/Et_2O = 5/5	First dissolved in DMSO, then dialyzed against MeOH

Notes: [a] The acronyms in the table, THF, MeOH, EtOH, Et_2O and DMSO, stand for tetrahydrofuran, methanol, ethanol, diethyl ether and dimethylsulfoxide, respectively. [b] In each reaction solvent, methanol was added incrementally, until there was no solid remaining, and the reaction mixture became translucent.

¹H-NMR Characterization of Polyampholytes with Mixed Side Chains

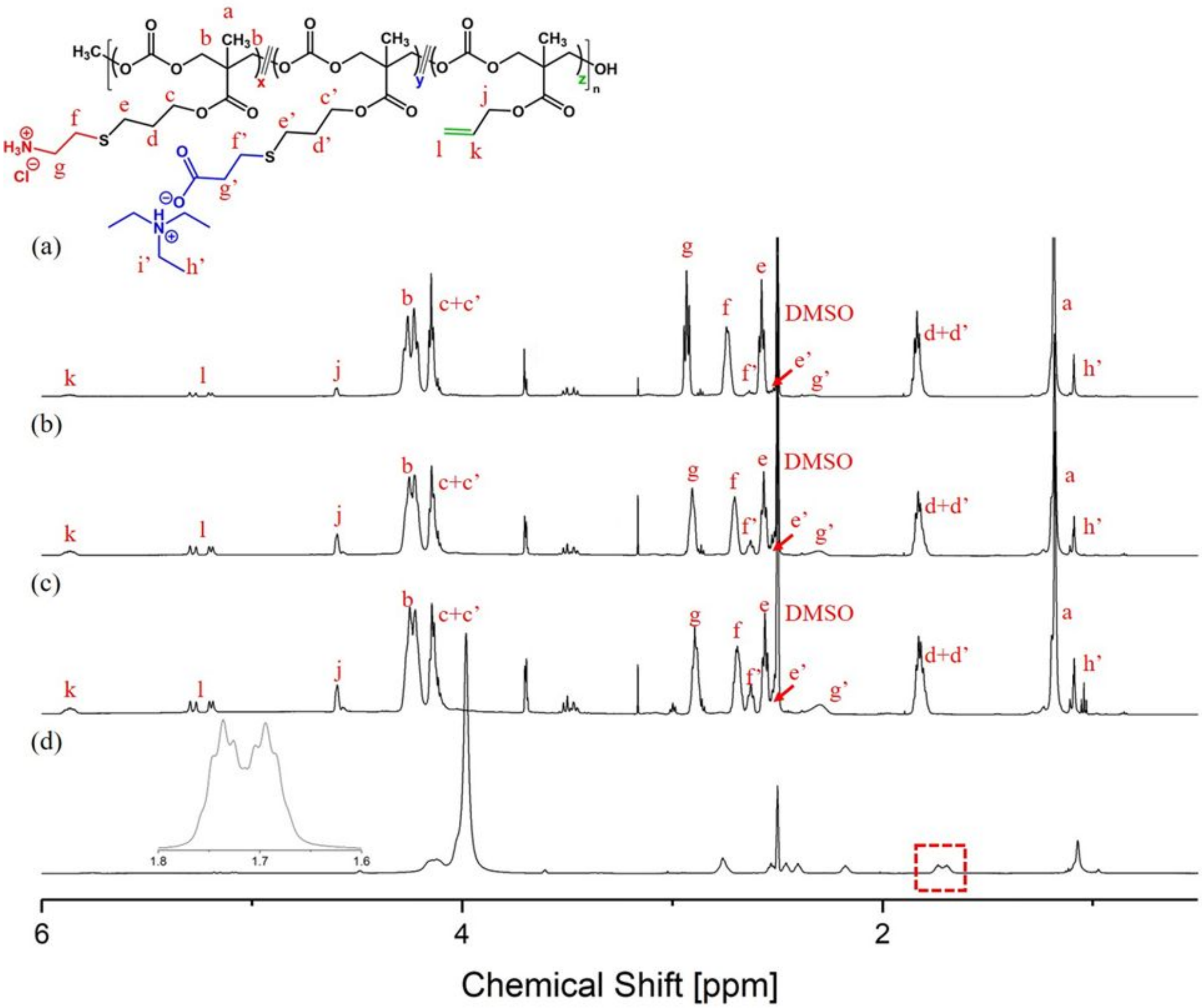

Figure S3. ¹H-NMR spectra of polyampholytes: (a) APC (+/−) 90/10; (b) APC (+/−) 80/20; (c) APC (+/−) 70/30; and (d) APC (+/−) 50/50, highlighting $\delta = 6 - 0.5$ ppm (600 MHz, in DMSO-d_6). For (d), the NMR solvent was saturated with LiCl to increase the resolution of the multiplet at around 1.8 ppm, as shown in the inset. This multiplet represents the peaks d and d' in the cation and anion side chains, respectively.

Time-Resolved DLS of APC polyelectrolytes in Aqueous Solution

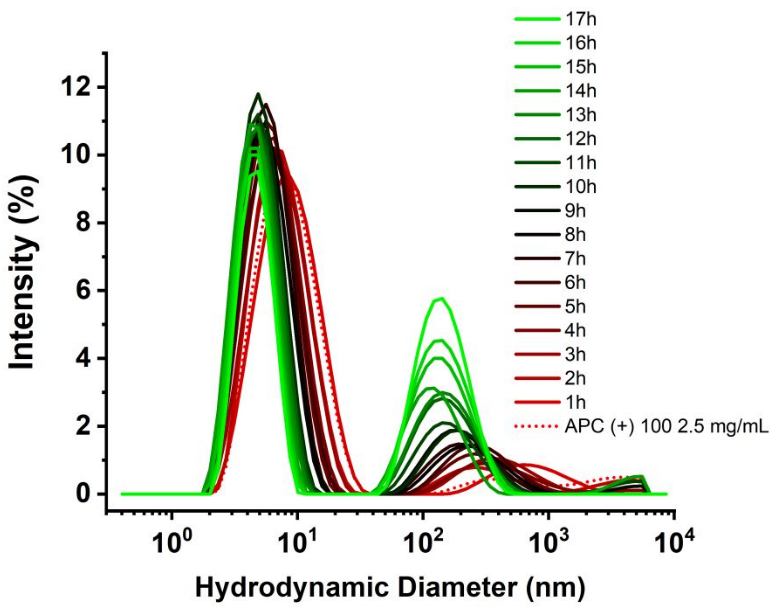

Figure S4. APC (+) 100 at 2.5 mg/mL, the same sample shown in Figure 4a of the main text, which was monitored by DLS every hour for overnight at 25 °C. The red dotted line represents the as-prepared solution (0 h).

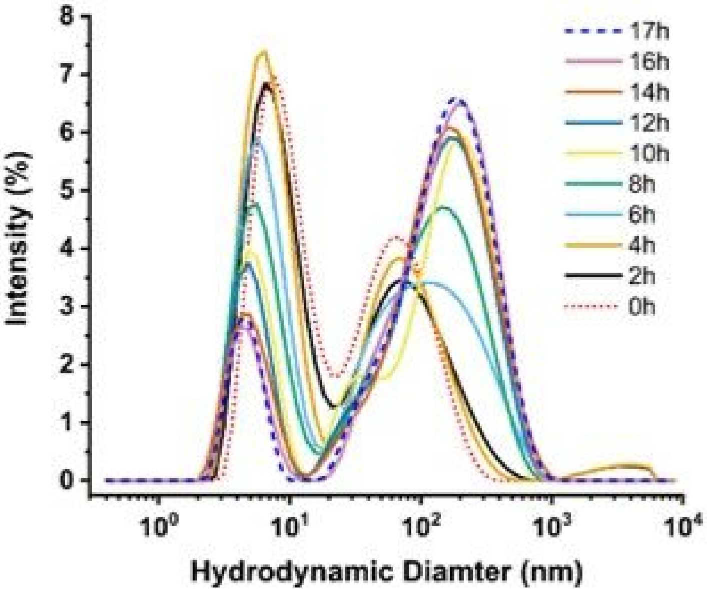

Figure S5. APC (+) 93 at 2.5 mg/mL monitored by DLS overnight at 25 °C.

Photographs of APC/Protein Complexation

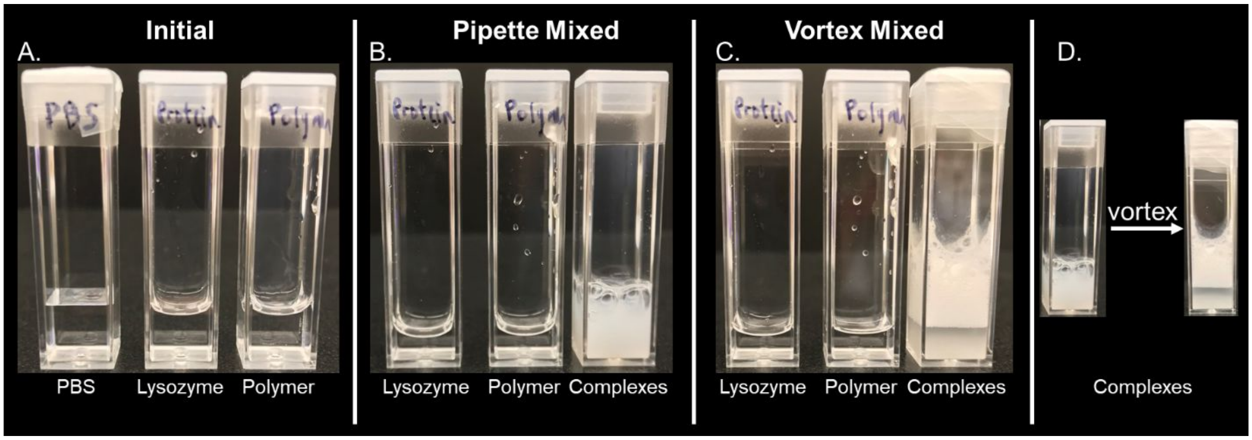

Figure S6. Photographs of lysozyme complexed with APC (-) 100. (A) Initial solutions prior to complexation. (B) Complex solution right after mixing (juxtaposed with the initial solutions). (C) Complex solution after prolonged vortex mixing (juxtaposed with the initial solutions). (D) Direct comparison between complex solutions before and after vortex mixing; in the right sample, sedimentation has already taken place. Photograph courtesy of the National Institute of Standards and Technology. Not subject to copyright in the United States.

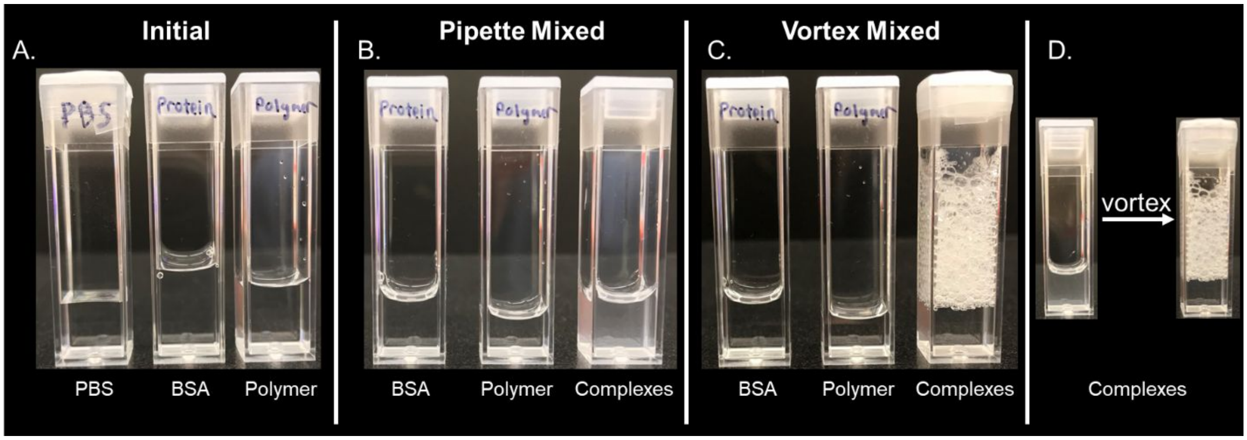

Figure S7. Photographs of BSA complexed with APC (+) 100. (A) Initial solutions prior to complexation. (B) Complex solution right after mixing (juxtaposed with the initial solutions). (C) Complex solution after vortex mixing (juxtaposed with the initial solutions). (D) Direct comparison between complex solutions before and after vortex mixing; in the right sample, the solution is completely clear and stable. Photograph courtesy of the National Institute of Standards and Technology. Not subject to copyright in the United States.

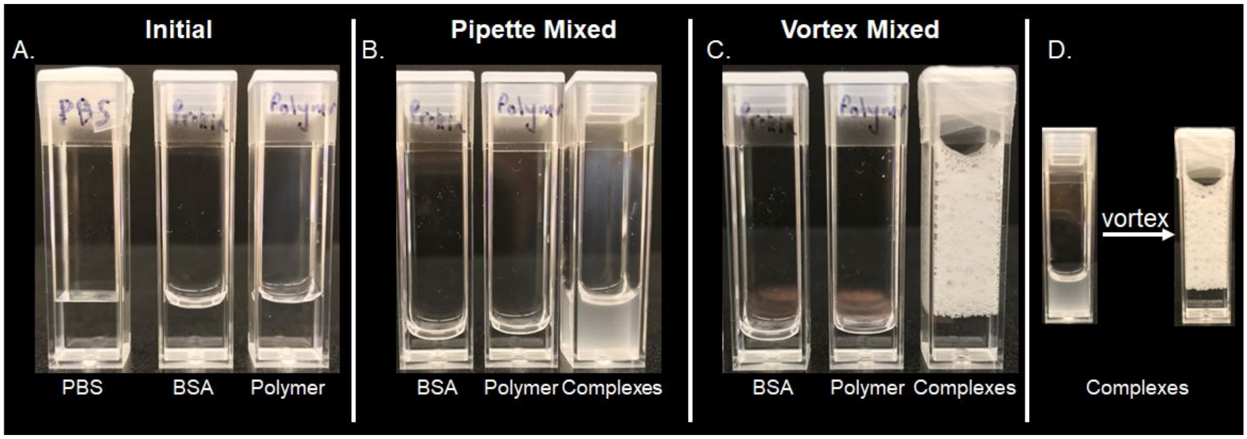

Figure S8. Photographs of BSA complexed with APC (+) 93. (A) Initial solutions prior to complexation. (B) Complex solution right after mixing (juxtaposed with the initial solutions). (C) Complex solution after vortex mixing (juxtaposed with the initial solutions). (D) Direct comparison between complex solutions before and after vortex mixing; the complex solution before vortex mixing (left) is apparently more turbid than that in Figure S7. Photograph courtesy of the National Institute of Standards and Technology. Not subject to copyright in the United States.

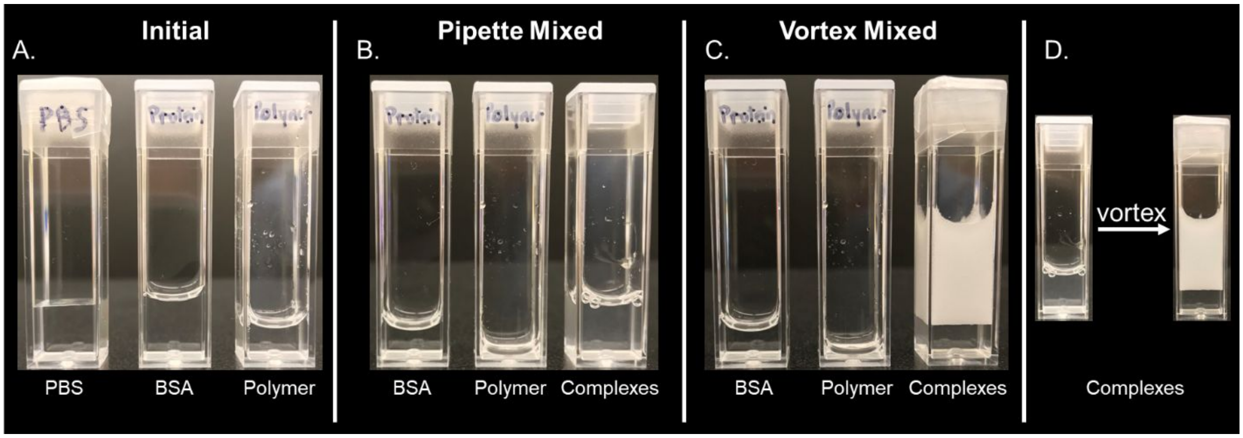

Figure S9. Photographs of BSA complexed with APC (+/-) 90/10. (A) Initial solutions prior to complexation. (B) Complex solution right after mixing (juxtaposed with the initial solutions). (C) Complex solution after vortex mixing (juxtaposed with the initial solutions). (D) Direct comparison between complex solutions before and after vortex mixing; the turbidity of the solution does not change dramatically before and after. Photograph courtesy of the National Institute of Standards and Technology. Not subject to copyright in the United States.

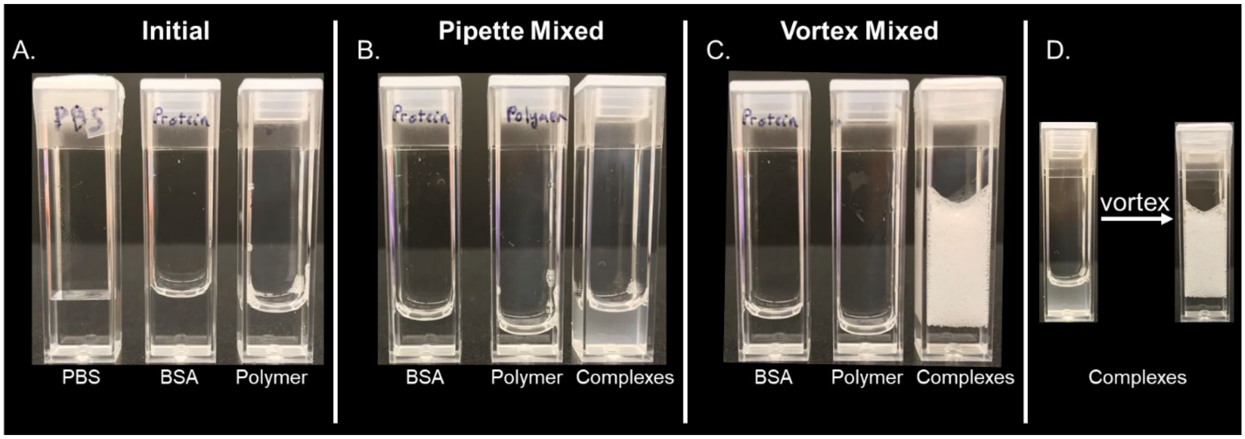

Figure S10. Photographs of BSA complexed with APC (+/-) 80/20. (A) Initial solutions prior to complexation. (B) Complex solution right after mixing (juxtaposed with the initial solutions). (C) Complex solution after vortex mixing

(juxtaposed with the initial solutions). (D) Direct comparison between complex solutions before and after vortex mixing; vortexing helps decrease the solution turbidity, as in the case of Figure S7 and S8. Photograph courtesy of the National Institute of Standards and Technology. Not subject to copyright in the United States.

SANS of APC (+) 100 and 93 in PBS-D_2O

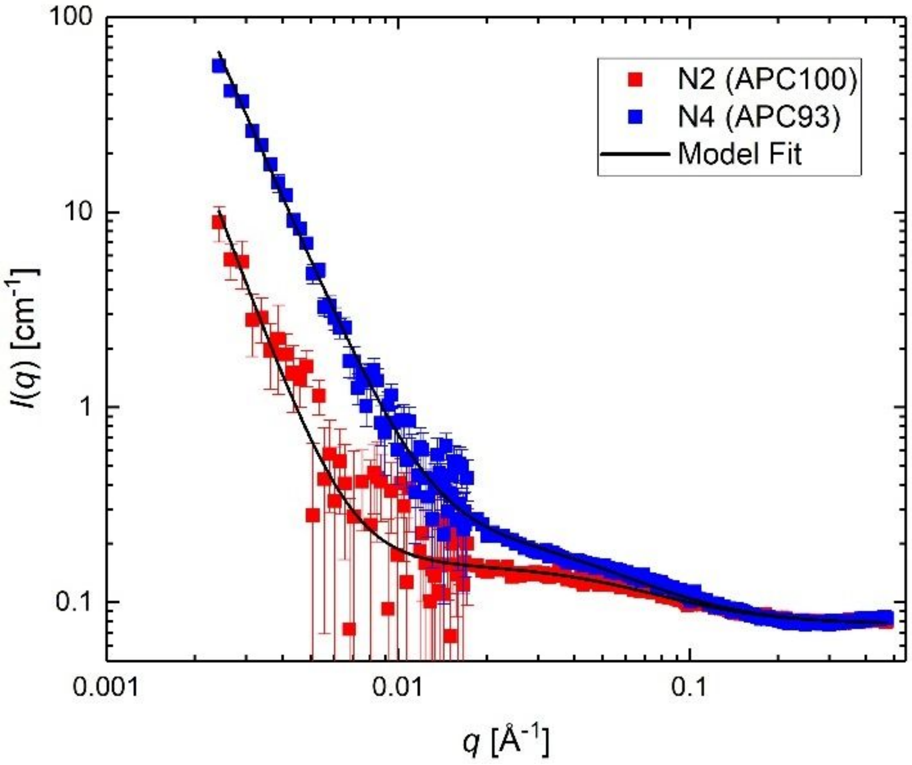

Figure S11. SANS of APC (+) 100 (red symbols) and APC (+) 93 (blue symbols) in PBS-D_2O. The black lines are best fit to eq. 1.

The SANS data of APC (+) 100 and APC (+) 93 in PBS-D_2O are shown in Figure S11. Notably, salt suppresses the correlation peaks as in Figure 6 of the main text. At low q, $I(q) \sim q^{-d}$ was used to model the upturn; at high q, the curves are best fit to the polydisperse Debye model, which assumes a Schulz-Zimm molecular weight distribution of the polymer. The overall model function reads:

$$I(q) = Aq^{-d} + B\frac{2[(1+Ux)^{-1/U} + x - 1]}{(1+U)x^2} + C \quad \text{(eq. 1)}$$

where $x = q^2 R_g^2/(1+2U)$, and $U = M_w/M_n - 1$. In the fitting, M_w/M_n is fixed at 1.38. The fitting results are summarized in Table S2.

Table S2. SANS Fitting Result of APC (+) 100 and 93.

Sample	d [-]	R_g [Å]
APC (+) 100	4.0 ± 0.4	26.3 ± 0.7
APC (+) 93	3.4 ± 0.1	32.8 ± 1.4

Estimate of Charge Density

Table S3. Charge characteristics of APC polyelectrolytes and proteins used in the complexation.

	concentration in complex (mg/mL)	molar mass (kg/mol)	DP of APC precursor	Net charge per polymer chain or protein	Molar net charge per gram (mol/kg) [d]
Lysozyme	2.5	14.4	–	+9 [b]	+0.62
APC (-) 100		29.7 [a]	73	−73 [c]	−2.46
BSA		66.4	–	−17 [b]	−0.26
APC (+) 93		24.4 [a]	80	+74 [c]	+3.03
APC (+) 100		22.9 [a]	73	+73 [c]	+3.19

Notes: [a] Calculated based on the degree of polymerization (DP) and NMR functionalization of each polymer. [b] Calculated by the difference between the positively charged amino acids K (lysine) and R (arginine), and the negatively charged D (aspartic acid) and E (glutamic acid), in their amino acid sequences. [c] Calculated by multiplying the DP with the charge fraction. [d] Calculated by the net charge divided by the molar mass.

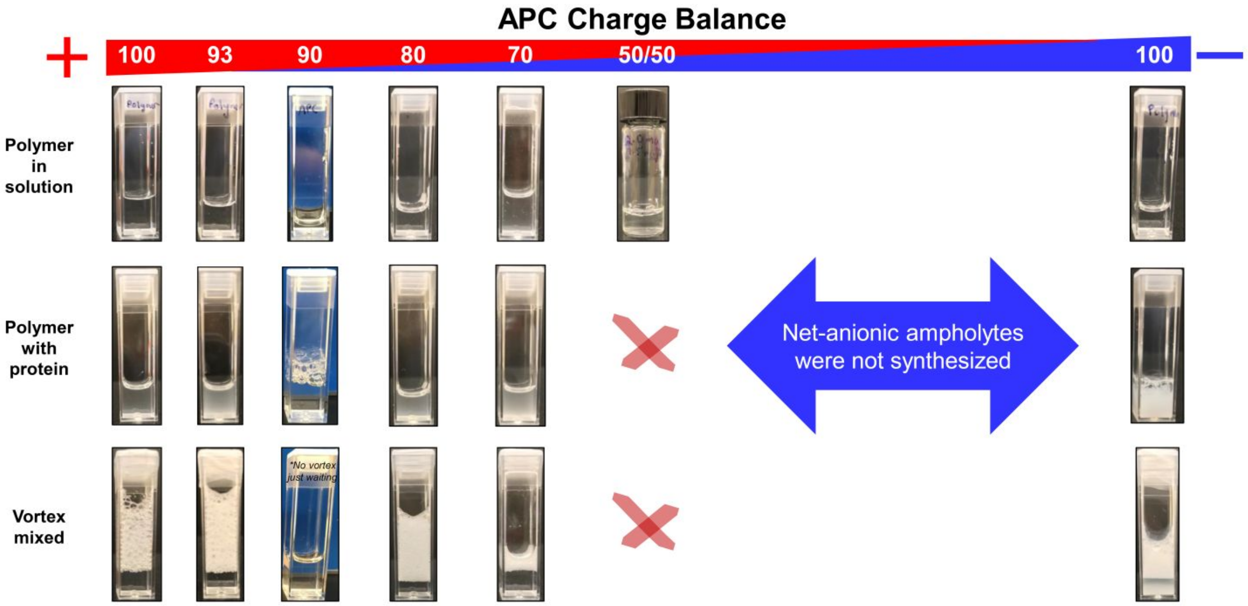

Figure S12. Conceptual summary of polymer charge balance impacting polymer solution, complexation, and response to mixing/processing with model proteins. APC (+/-) 50/50 pictured in the vial is insoluble where polymer solids adhere to the vial sides after vortex mixing. Red X's indicate that the polymer is not used for in-depth protein complexation studies. Photograph courtesy of the National Institute of Standards and Technology. Not subject to copyright in the United States.

Figure S12 combines qualitative observations of solution/complexation behavior and maps them onto a net-charge spectrum that includes all charged polymers in this report. The top row of pictures qualitatively depicts polyelectrolyte solubility in biologically relevant PBS at pH = 7.4. These findings show that polyelectrolytes at the ends of the spectrum have desirable solubility and solution properties which will inform the design of future polymers for protein binding applications. The middle and bottom row of pictures depict the protein complexation behavior and response to processing/mixing conditions for each of the polymer. Again, the mixture of APC (+/-) 90/10 and BSA stands out clearly from the other polyelectrolytes.

SANS on BSA with APC (+) 100 and 93 in PBS-D_2O

The structure of bovine serum albumin was originally found to have a prolate ellipsoid shape with a semi-major axis of 70 Å and semi-minor axis of 20 Å by small-angle neutron scattering.[1] This was consistent with earlier sedimentation equilibrium measurements.[2] These sources justified the present selection of an ellipsoid model applied for different solution and buffer conditions.

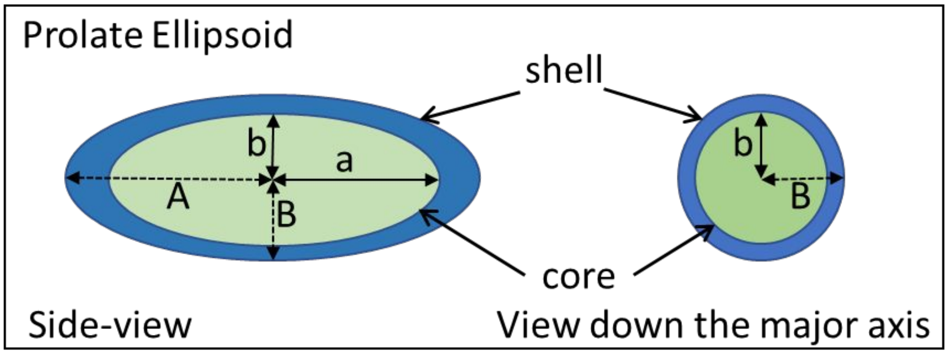

Figure S13. Sectioned schematic of side and end-on view of a model core-shell prolate ellipsoid with semi-major and semi-minor axis.

The main text shows SANS data of the BSA. The ellipsoid model was used without modification in the NIST Igor Pro SANS analysis software.[3] The main parameters of interest are the semi-major (a) and semi-minor (b) radii of the BSA prolate ellipsoid (Figure S13) and the effects of polymer complexation to BSA. Additional parameters are needed or may be estimated including the particle concentration and scattering length density (ρ) associated with the ellipsoid model. In this case the ρ_c of BSA was fixed to 3.8 $\times10^{-6}$ Å^{-2}, consistent with prior studies[1], ρ_s of the D_2O PBS buffer was 6.3 $\times10^{-6}$ Å^{-2}. The least squares curve fitting results are shown in Table 2 which includes a flat scattering background due to incoherent scattering arising from primarily the protons from the BSA after the D_2O buffer scattering was subtracted from the data. The uncertainty (±) represent one standard deviation of the fit.

Table S4. SANS curve fitting parameters[a]

	BSA	BSA+ APC(+)100	BSA+ APC(+)93
Scale factor = particle fraction	(4.0±0.04)$\times10^{-3}$	(1.62 ±0.03)$\times10^{-3}$	(1.69 ±0.03)$\times10^{-3}$
major core radius, a (Å)	60.3 ± 0.7	60.3	60.3
minor core radius, b (Å)	23.5 ± 0.2	23.5	23.5
major shell radius, A (Å)	-	86.5 ±0.9	83.76 ±0.8
minor shell radius, B (Å)	-	23.92 ±0.066	23.5 ±0.06
ρ_c (Å^{-2})	3.8 $\times10^{-6}$	3.8 $\times10^{-6}$	3.8 $\times10^{-6}$
ρ_{sh} (Å^{-2})	-	1.0$\times10^{-6}$	1.0$\times10^{-6}$
ρ_s (Å^{-2})	6.3 $\times10^{-6}$	6.3 $\times10^{-6}$	6.3 $\times10^{-6}$
background (cm^{-1})	0.0154 ±0.00007	0.021958 ±0.00006	0.01558 ±0.00005

[a]Parameters that were fit are shown with uncertainties (±) that represent one standard deviation to the model fit.

Our results differ slightly from the literature for BSA with semi-axes of 60 Å and 23 Å. We attribute these differences to the different D_2O buffers used between the studies as well as a convoluted effect that may be caused by BSA dimers. The BSA fit result could not recover the feature between q = 0.15 Å^{-1} to 0.25 Å^{-1}. This may be caused by the presence of BSA dimers,

which were not removed. This feature appears in the BSA as well as the complex and therefore not associated with the polymer and further attempts to include would only lead to more parameters. We then fix the BSA structure and contrast factor as the core of the core-shell ellipsoid model and fit the BSA with the expected bound APC polycations as shown in Table S4 using an estimated ρ_{sh} for the APC polymer as 1.0×10^{-6} Å^{-2}. Figure S13 shows the basic geometric factors defined along two views of the prolate ellipsoid.

The fit results are summarized in Table S4 and in Table 2. The initial major core radius was held fixed at 60.3 Å and the major shell thickness resulted in 86.5 Å, a 26.2 Å increase. Interestingly, with the initial minor core radius held fixed at 23.5 Å, there was little change in the minor shell radius after complexation with APC(+)100. Similar results are observed with APC(+)93.

The increase in the radius of the shell relative to the core represents a change due to the polycation binding. We assume there are negligible free polycation chains in the solution following the complexation with BSA, as the scattering data are best fit by a single model of core-shell ellipsoid, rather than a combined model of ellipsoid and Gaussian coil.

The main equations for the prolate ellipsoid core-shell model may be found in the original sources[4] and the NIST Igor Pro data analysis software package.[3] We reproduce the key equations that do not include the structure factor, $S(q)$, contributions that consider interparticle interactions and correlations due to the dilute solutions used and high salt concentrations that screen the long range electrostatic interactions. The scattered intensity $I(q)$ involves an orientational average of the single-particle form factor, $|F(q)|^2$ as a function of the integration variable (α), with prefactors of the particle volume fraction, ϕ, volume of the particle V_t, and a background (I_b) from solvent scattering and incoherent scattering arising primarily from the protons,

$$I(q) = \frac{\phi}{V_t}\int_0^1 |F(q,\alpha)|^2 d\alpha + I_b \qquad \text{Eq. 2}$$

The volume fraction of the particles is inter-related to the number density (n) of particles by $\phi = nV_t$, which must be considered when using the Igor Pro Models as input or fitting the scale prefactor concentration.

The amplitude of the scattering by a core-shell ellipsoid is defined by,

$$F(q,\alpha) = (\rho_c - \rho_{sh})V_c\left[3\frac{j_1(u_c)}{u_c}\right] + (\rho_{sh} - \rho_s)V_t\left[3\frac{j_1(u_t)}{u_t}\right] \quad . \qquad \text{Eq. 3}$$

The neutron contrast prefactors represent the scattering length density difference between core and shell in the first term and shell and solvent in the second term. The volumes of the core (V_c) and total core-shell (V_t) ellipsoid are defined by, Eq 4 and Eq 5, respectively, with semi-axis defined earlier and shown in Figure S13.

$$V_c(a,b) = \frac{4\pi}{3}ab^2 \qquad \text{Eq. 4}$$

$$V_t(A,B) = \frac{4\pi}{3}AB^2 \qquad \text{Eq. 5}$$

The spherical Bessel, j_1,

$$j_1(u_i) = (sin\, u_i - u_i cos u_i)/u_i^2 \qquad \text{Eq. 6}$$

with variable u_i are defined for different index, i, for the core and total prolate ellipsoid by

$$u_c = q\left[a^2\alpha^2 + b^2(1 - \alpha^2)\right]^{1/2} \qquad \text{Eq. 7}$$

and

$$u_t = q\left[A^2\alpha^2 + B^2(1 - \alpha^2)\right]^{1/2} \qquad \text{Eq. 8}$$

over the common integration variable, $\alpha = \cos w$, where w is the orientation angle between the scattering vector q and semi-major axis a. As can be seen many of the variables appear as products and therefore are highly correlated. These correlations were minimized by keeping many of the parameters that may be estimated based upon the chemical composition or literature results, such

as scattering length densities. In the present case, the concentration was also fit which can lead to correlations to the geometric parameters. However, once the geometric parameters for the BSA core were fixed the only fit parameters would be the semi-axis of the core-shell ellipsoid and prefactor, was very close between the two polymers consistent with the sample preparation. The small contribution from the incoherent background after subtracting the solvent scattering is another parameter.

Estimation of effect of methanolysis during dialysis by aqueous size-exclusion chromatography (SEC)

Aqueous SEC was performed with a Waters 1515 Isocratic HPLC Pump equipped with a Waters 717 Autosampler, Waters 2487 Dual λ Absorbance Detector (264 nm), and Waters 2414 Refractive Index Detector in series with Eprogen CATSEC columns (CATSEC-1000 guard, CATSEC-100, CATSEC-300, and CATSEC-1000) (packing media contains a proprietary polymeric bonding chemistry on spherical silica support). The aqueous SEC eluent was 1% volume/volume of acetic acid and 0.1 molar lithium bromide in water and run at a flow rate of 0.3 mL/minute. All components of the instrument were at room temperature except for the refractive index (RI) detector at 30 ^{o}C and the columns at 60 ^{o}C. The SEC instrument was interfaced using Waters Breeze v3.30 software. Relative molar masses and molar mass distributions were determined using calibration curves from narrow poly(2-vinyl pyridine) (PVP) (4,800 Da to 539,000 Da) calibrants.

This SEC provides relative molar mass which differs from the SEC in THF with absolute molar mass measurement via light scattering detection. Aqueous SEC traces for freshly prepared from the solid APC(+)93 purified by precipitation and no methanol dialysis is similar to the APC(+)100 that was purified by both precipitation and methanol dialysis. We provide this as the test that the polymers, as freshly prepared, are not substantially affected by methanolysis after post-

polymerization modification. The relative mass-average molar mass for the APC(+)93 is Mw = 17,200 Da with 1.29 polydispersity and APC(+)100 was 11,800 Da with 1.16 polydispersity, such data are not comparable to GPC in THF with absolute molar mass via light scattering detection, but provide confidence for the series of polymers to enable a net-charge study.

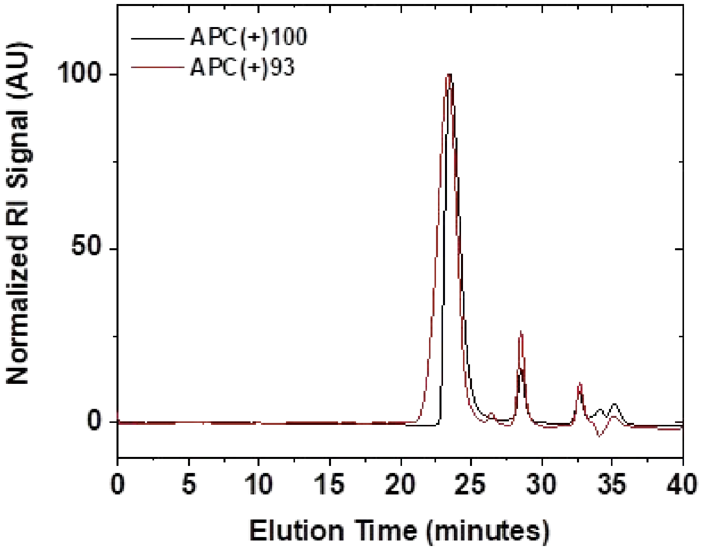

Figure S14. Aqueous SEC of APC(+)93 and APC(+)100 showing the primary elution peak and polydispersity determined from single injection.

References

(1) Bendedouch, D.; Chen, S. Structure and Interparticle Interactions of Bovine Serum-Albumin in Solution Studied by Small-Angle Neutron-Scattering. *J. Phys. Chem.* **1983**, *87* (9), 1473–1477. https://doi.org/10.1021/j100232a003.

(2) Squire, P.; Moser, P.; Okonski, C. Hydrodynamic Properties of Bovine Serum Albumin Monomer and Dimer. *Biochemistry* **1968**, *7* (12), 4261. https://doi.org/10.1021/bi00852a018.

(3) Kline, S. Reduction and Analysis of SANS and USANS Data Using IGOR Pro. *J. Appl. Crystallogr.* **2006**, *39* (6), 895–900.

(4) Berr, S. Solvent Isotope Effects on Alkyltrimethylammonium Bromide Micelles as a Function of Alkyl Chain-Length. *J. Phys. Chem.* **1987**, *91* (18), 4760–4765. https://doi.org/10.1021/j100302a024.
